# Multimodality approach to the nipple-areolar complex: a pictorial review and diagnostic algorithm

**DOI:** 10.1186/s13244-020-00896-1

**Published:** 2020-08-05

**Authors:** Javier del Riego, Mireia Pitarch, Clara Codina, Laura Nebot, Francisco J. Andreu, Oscar Aparicio, Alexandra Medina, Amaya Martín

**Affiliations:** 1 Department of Radiology, Women’s Imaging, UDIAT Centre Diagnòstic, Parc Taulí Hospital Universitari, Institut d’Investigació i Innovació Parc Tauli I3PT, Univertitat Autònoma de Barcelona, 1 Parc Tauli, Sabadell, Barcelona, Spain; 2 Women’s Imaging, Grup Duran Diagnòstic per la Imatge, Sabadell, Barcelona, Spain; 3 Department of Pathology, Parc Taulí Hospital Universitari, Institut d’Investigació i Innovació Parc Tauli I3PT, Univertitat Autònoma de Barcelona, Barcelona, Spain; 4 Department of Pathology, Atryshealth S.L, Barcelona, Spain; 5 Department of Surgery, Parc Taulí Hospital Universitari, Institut d’Investigació i Innovació Parc Tauli I3PT, Univertitat Autònoma de Barcelona, Barcelona, Spain; 6 Department of Gynecology and Obstetrics, Parc Taulí Hospital Universitari, Institut d’Investigació i Innovació Parc Tauli I3PT, Univertitat Autònoma de Barcelona, Barcelona, Spain

**Keywords:** Nipple-areolar complex, Breast disease, Mammography, Sonography, Contrast-enhanced magnetic resonance imaging

## Abstract

The anatomic and histologic characteristics of the nipple-areolar complex make this breast region special. The nipple-areolar complex can be affected by abnormal development and a wide spectrum of pathological conditions, many of which have unspecific clinical and radiological presentations that can present a challenge for radiologists. The nipple-areolar complex requires a specific imaging workup in which a multimodal approach is essential. Radiologists need to know the different imaging modalities used to study the nipple-areolar complex, as well as their advantages and limitations. It is essential to get acquainted with the acquisition technique for each modality and the spectrum of findings for the different conditions. This review describes and illustrates a combined clinical and radiological approach to evaluate the nipple-areolar complex, emphasizing the findings for the normal morphology, developmental abnormalities, and the most common benign and malignant diseases that can affect this region. We also present a diagnostic algorithm that enables a rapid, practical approach to diagnosing condition involving the nipple-areolar complex.

## Key points

- Appropriate techniques are essential to avoid pitfalls in the nipple-areolar complex.
- Diagnosis requires joint assessment of clinical and multimodality imaging findings.
- Inversion differs from retraction; both occur in benign and malignant conditions.
- Inflammatory/infectious conditions require ultrasound follow-up study in 4 to 6 weeks.
- In case of doubt, always biopsy.

## Background

The nipple-areolar complex is a region of the breast that has unique characteristics. It is composed of different cells and specific tissues whose main function is to facilitate the drainage and secretion of breast milk during lactation [1]. A wide variety of abnormal conditions can affect the nipple-areolar complex, including developmental abnormalities, benign processes (e.g., inflammation, infection, tumors), and invasive and noninvasive cancers [2–5].

Many of these conditions have nonspecific clinical and radiological presentations that can delay diagnosis, so evaluating the nipple-areolar complex represents a challenge for radiologists. A detailed history and clinical examination are essential to guide the radiological management of the nipple-areolar complex. Recognizing the different clinical signs that can manifest in the nipple-areolar complex (e.g., skin involvement, pathological nipple discharge, retraction, inversion, palpable mass, etc.) is the first step in ensuring effective radiological management.

Imaging studies play an important role in diagnosing nipple-areolar complex conditions. Since this is a mobile, superficial region, it requires a specific approach to imaging evaluation. A meticulous radiological technique is fundamental to avoid artifacts and pitfalls. Furthermore, a multimodality approach is essential. The retroareolar region is difficult to evaluate in mammograms, so disease often goes undetected. For this reason, other techniques such as ultrasound (US) and even magnetic resonance imaging (MRI) are necessary to reach the diagnosis.

Lastly, it is very important to predict tumor involvement in the nipple-areolar complex before surgery. On the one hand, knowledge of nipple-areolar complex involvement is fundamental for staging disease (prognosis); on the other hand, thanks to improvements in breast-conserving techniques, it can be extremely helpful in planning the surgical management of breast cancer [6, 7].

In this review, we use a combined clinical/multimodal imaging approach for the nipple-areolar complex to describe and illustrate the singularities of the radiological techniques, the normal morphology, developmental abnormalities, and the main benign and malignant diseases. We discuss the characteristics of the different imaging techniques and provide guidance on how to avoid artifacts and pitfalls. Finally, we present a diagnostic algorithm for a rapid, practical approach to imaging to help ensure effective diagnosis.

## Anatomy and development

### Normal anatomy

**Fig. 1:**
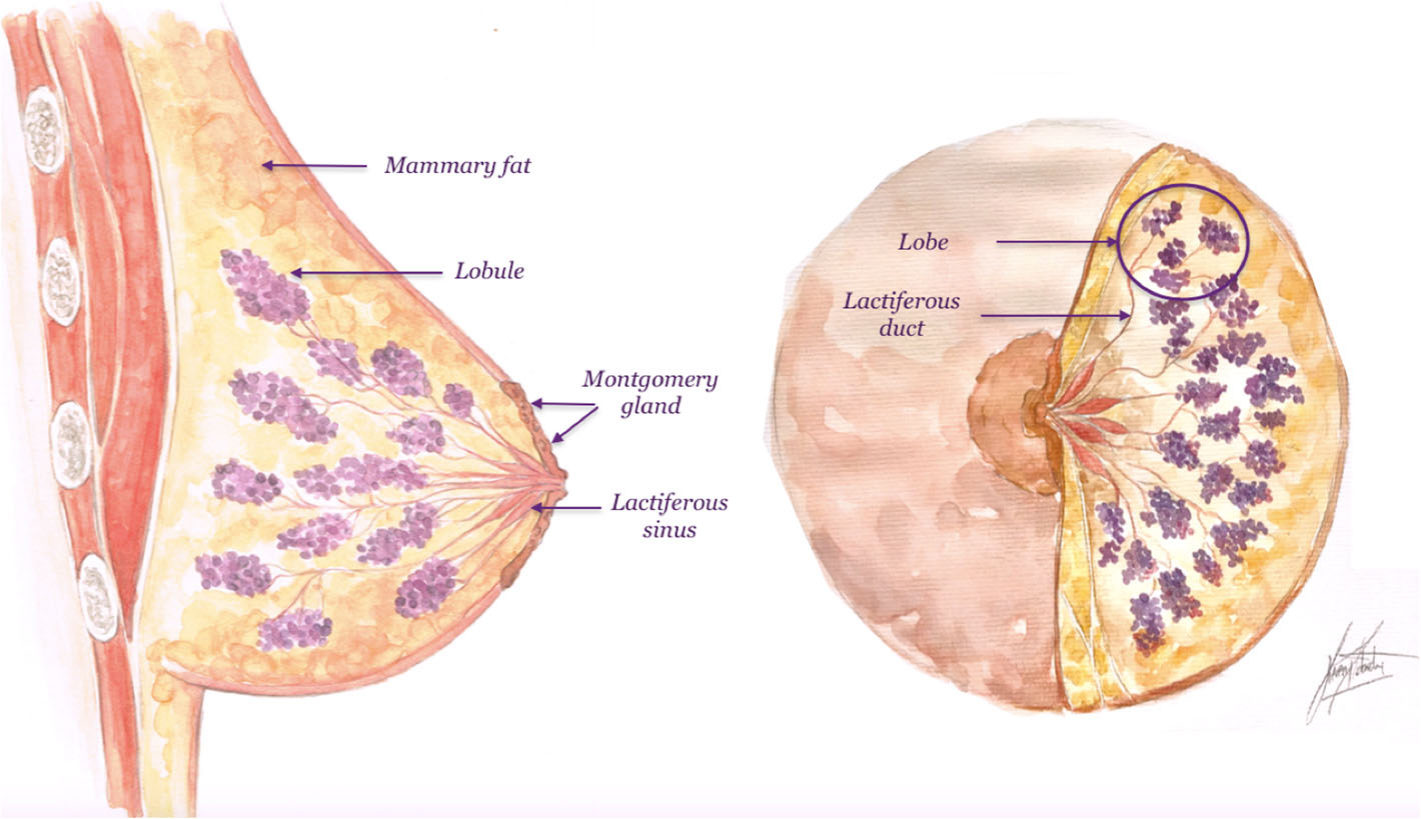
Anatomy of the nipple-areolar complex

The nipple-areolar complex is the pigmented area in the most prominent part of the breast where the lactiferous ducts draining the 15 to 20 lobes of the mammary gland converge [8]. These lobes are oriented radially toward the nipple, and each lobe is made up of several lobules (Fig. 1) [1]. Each lobule has a lactiferous duct that in turn branches and ends in the terminal ductal lobular unit (TDLU), which is the functional unit of the breast gland [8–10]. In the subareolar region, the ducts expand to form the lactiferous sinus [11]. The ducts then drain through 5 to 9 orifices in the nipple [10].

**Fig. 2:**
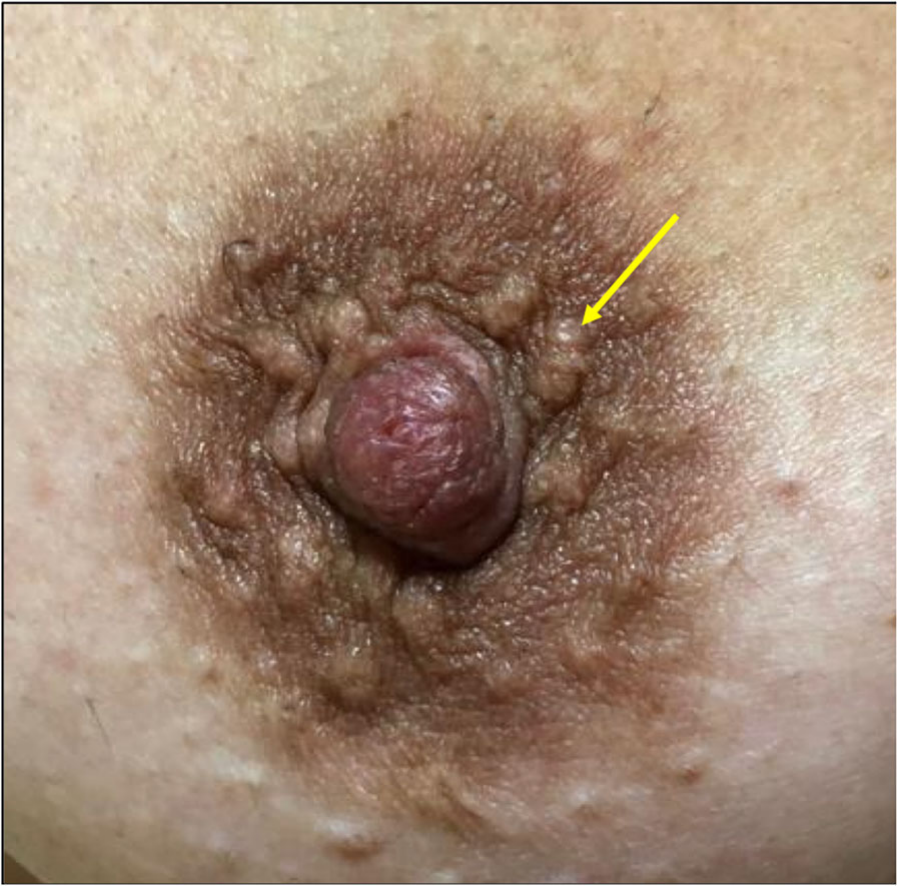
Morgagni tubercles. Photograph of a nipple-areolar complex. Note the small bumps in the skin (arrow)

The nipple-areolar complex is lined with stratified keratinized squamous epithelium [12]. This epithelium extends toward the inside of the orifices of the nipple ducts, which explains how a carcinoma located in the subareolar ducts can extend to the skin of the nipple [12, 13]. The small raised areas on the skin of the areola (1–2 mm) are called Morgagni tubercles (Fig. 2) [1]. The tubercles are the openings of the ducts of the Montgomery glands, modified sebaceous glands that are connected to small, rudimentary mammary glands and can therefore secrete milk [1]. These glands become more prominent during pregnancy and help lubricate the areola during lactation [1].

The nipple-areolar complex also contains numerous sensory nerve endings, smooth muscle fibers, pilosebaceous follicles, and a rich subareolar lymphatic plexus (Sappey plexus) [12, 13].

### Embryological development

**Fig. 3:**
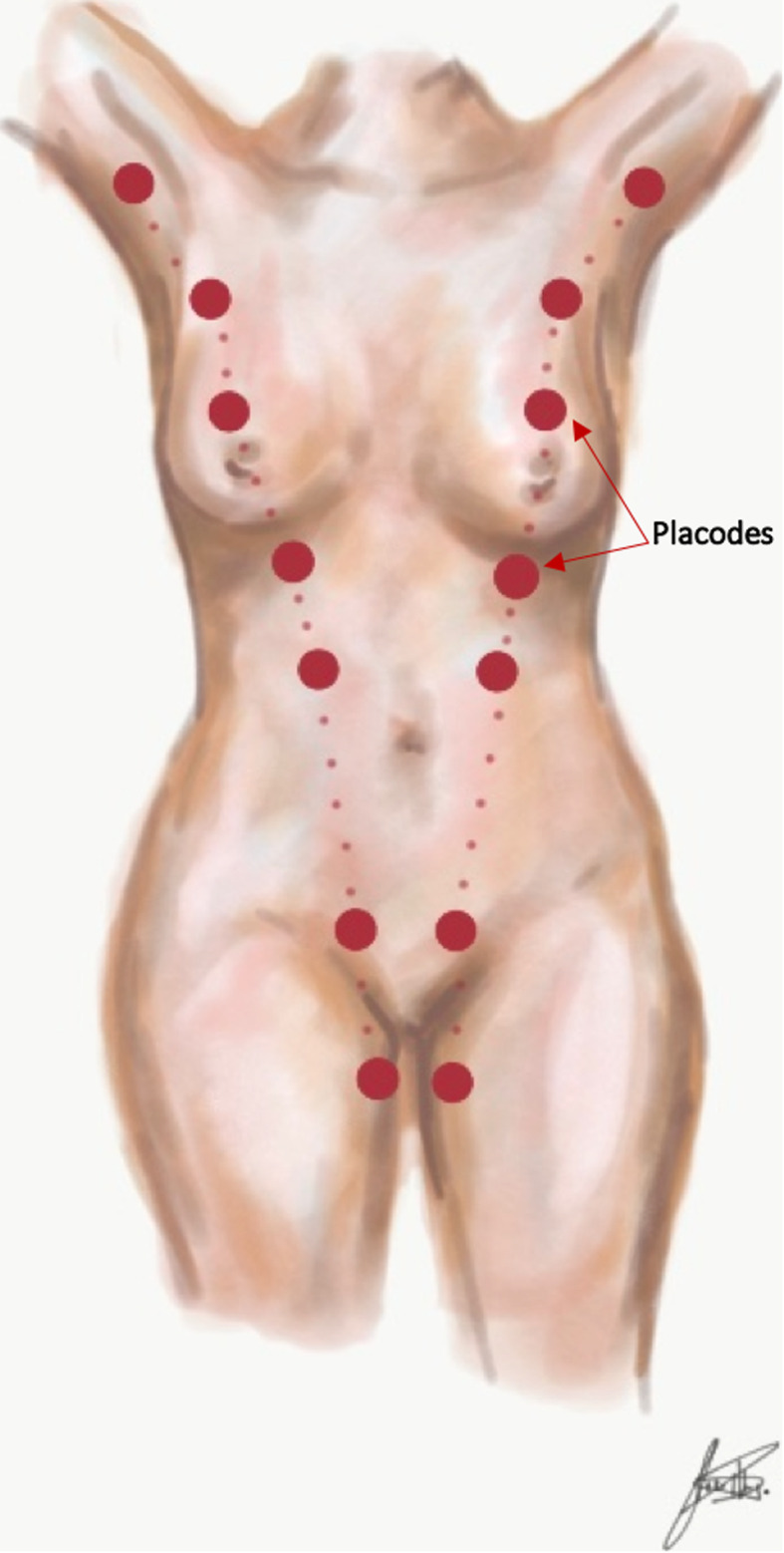
Milk lines. Placodes form along the milk lines, which extend from the axillae to the inguinal region (arrow)

Starting in the 5th or 6th week of gestation, the ectodermal ridges (also called milk lines) start to form on both sides of the anterior aspect of the embryo, running from the axillae to the inguinal region (Fig. 3) [11]. Placodes forming along these ridges will later invaginate, giving rise to several mammary buds [14, 15]. These mammary buds normally atrophy, except the ones located in the fourth intercostal space on either side, which will develop into the breasts [16]. Incomplete invagination of the mammary buds results in developmental abnormalities [2].

In the 12th through the 16th weeks of gestation, the mesenchymal cells differentiate into smooth muscle in the areola and nipple [9, 11]. Between the 32nd and 40th weeks, parenchymal differentiation results in the development and pigmentation of the nipple-areolar complex [9].

### Congenital anomalies

Developmental anomalies can be unilateral or bilateral and can involve the nipple, the breast, or both [2]. These anomalies are most common in the axillary region and in the inframammary fold, although they can arise at any point along the milk lines [11, 16].

**Fig. 4:**
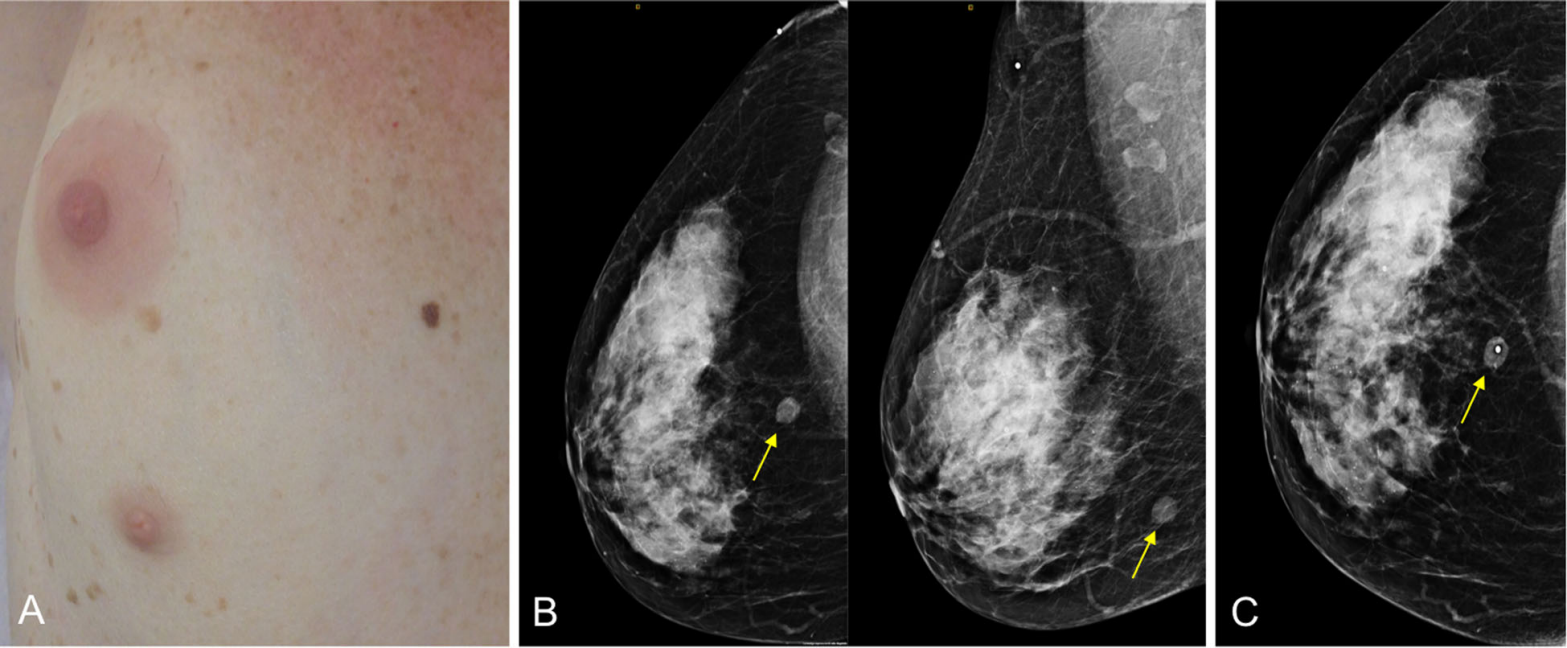
Developmental abnormalities. **a** Photograph of polythelia in the right breast of a 45-year-old woman. **b** Craniocaudal and mediolateral oblique 2D mammograms show a nodule with well-defined margins in the posterior region of the junction between the lower quadrants. **c** Repeat craniocaudal mammogram with a cutaneous marker confirms that the nodule corresponds to the accessory nipple

The most common anomaly is polythelia, the presence of a supernumerary nipple, which can be mistaken for a pigmented nevus on physical examination (Fig. 4) [2]. Rarely, fibroglandular tissue underlies the accessory nipple (polymastia) [9].

The congenital absence of the nipple-areolar complex (athelia) is usually accompanied by the absence of breast tissue (amastia) [2, 17]; more rarely, the nipple is present but the mammary gland is absent (amazia) [2]. Underdevelopment of the breast is called hypoplasia [18]. Sometimes, these anomalies are found together with other developmental anomalies and can even form part of syndromes, such as Poland syndrome [19].

The tuberous breast deformity is a rare developmental abnormality that is characterized by glandular hypoplasia, deficiency in the circumferential skin envelope at the base of the breast, asymmetry, and herniation of fibroglandular tissue in the areolar region [20].

## Imaging techniques

Due to the anatomic complexity, superficial location, and mobility of the nipple-areolar complex, this area of the breast requires special considerations in imaging tests.

### Mammography

For the evaluation of the retroareolar area, mammography is less sensitive than ultrasound, partly owing to the greater density of this complex anatomic region and partly owing to technical difficulties due to the mobility of this part of the breast [21, 22].

**Fig. 5:**
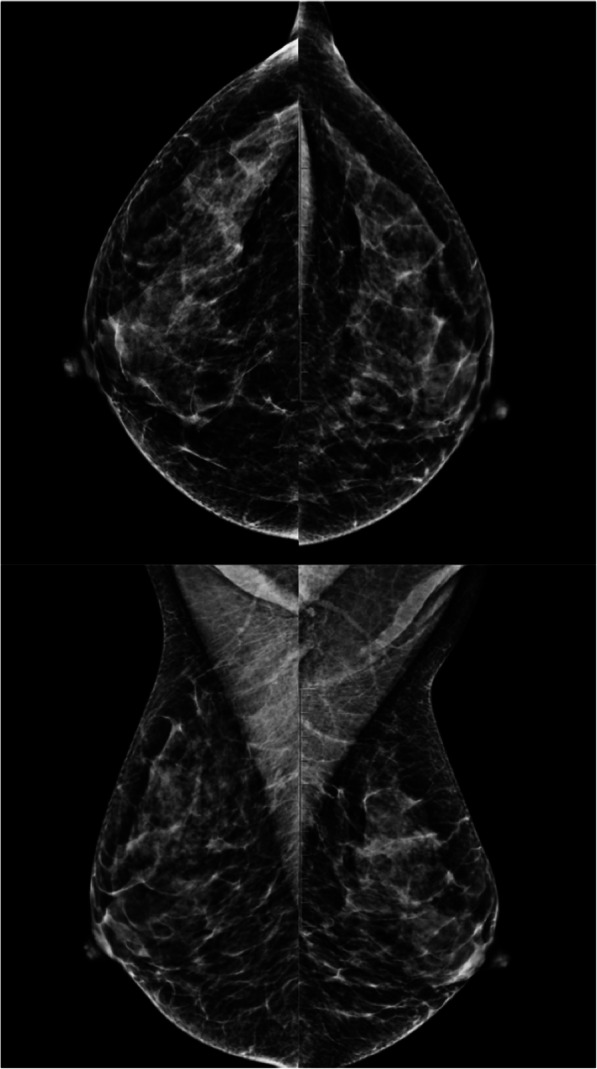
Mammography positioning. Craniocaudal and mediolateral oblique 2D mammograms show the nipples are perfectly tangential

**Fig. 6:**
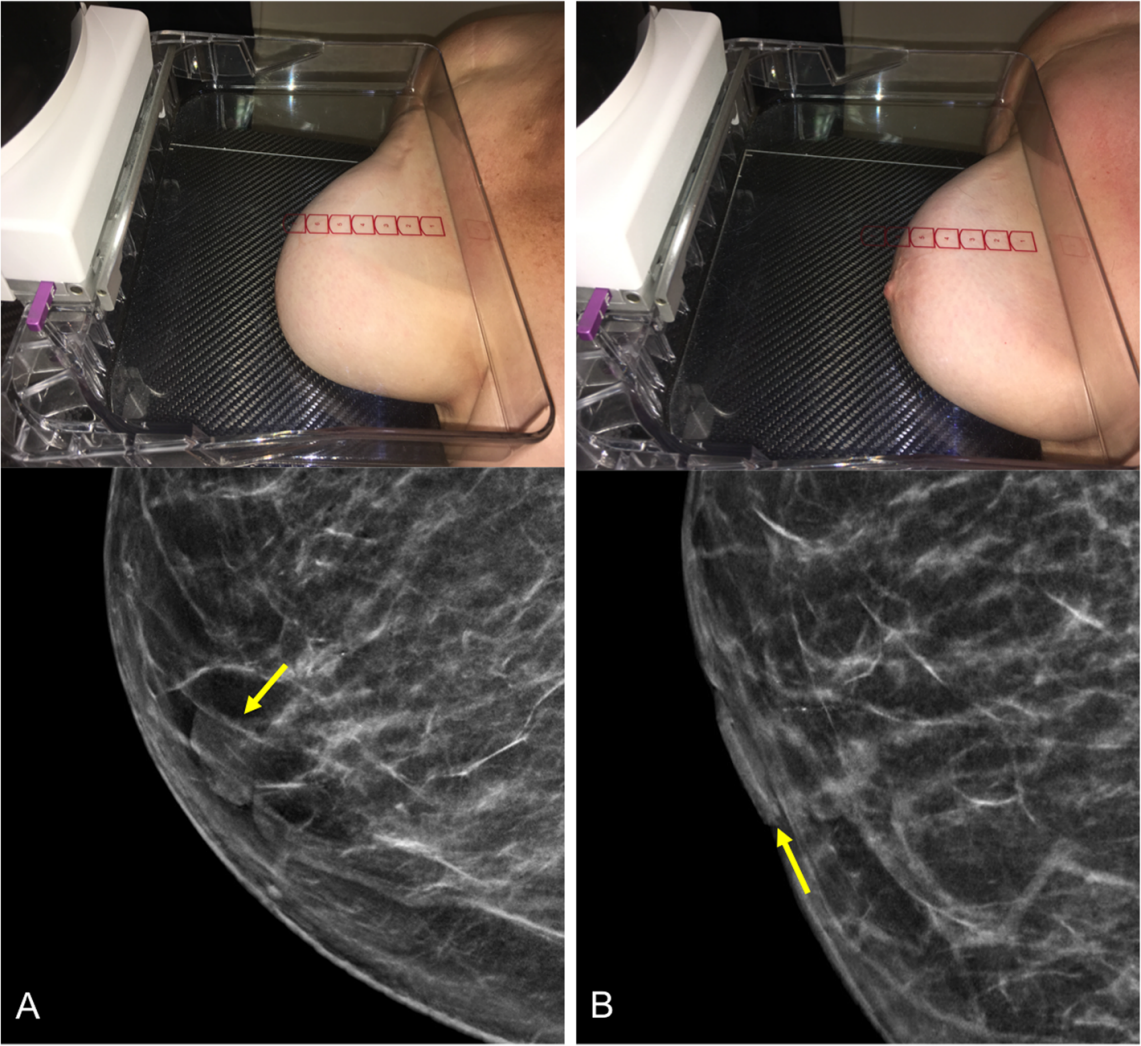
Projection centered in the anterior region. A 56-year-old woman with voluminous breasts. **a** Craniocaudal 2D mammogram: the nipple is not tangential and is hidden in the lower part of the breast, producing a false image of a nodule (arrow). **b** Repeat craniocaudal view with the nipple tangential

**Fig. 7:**
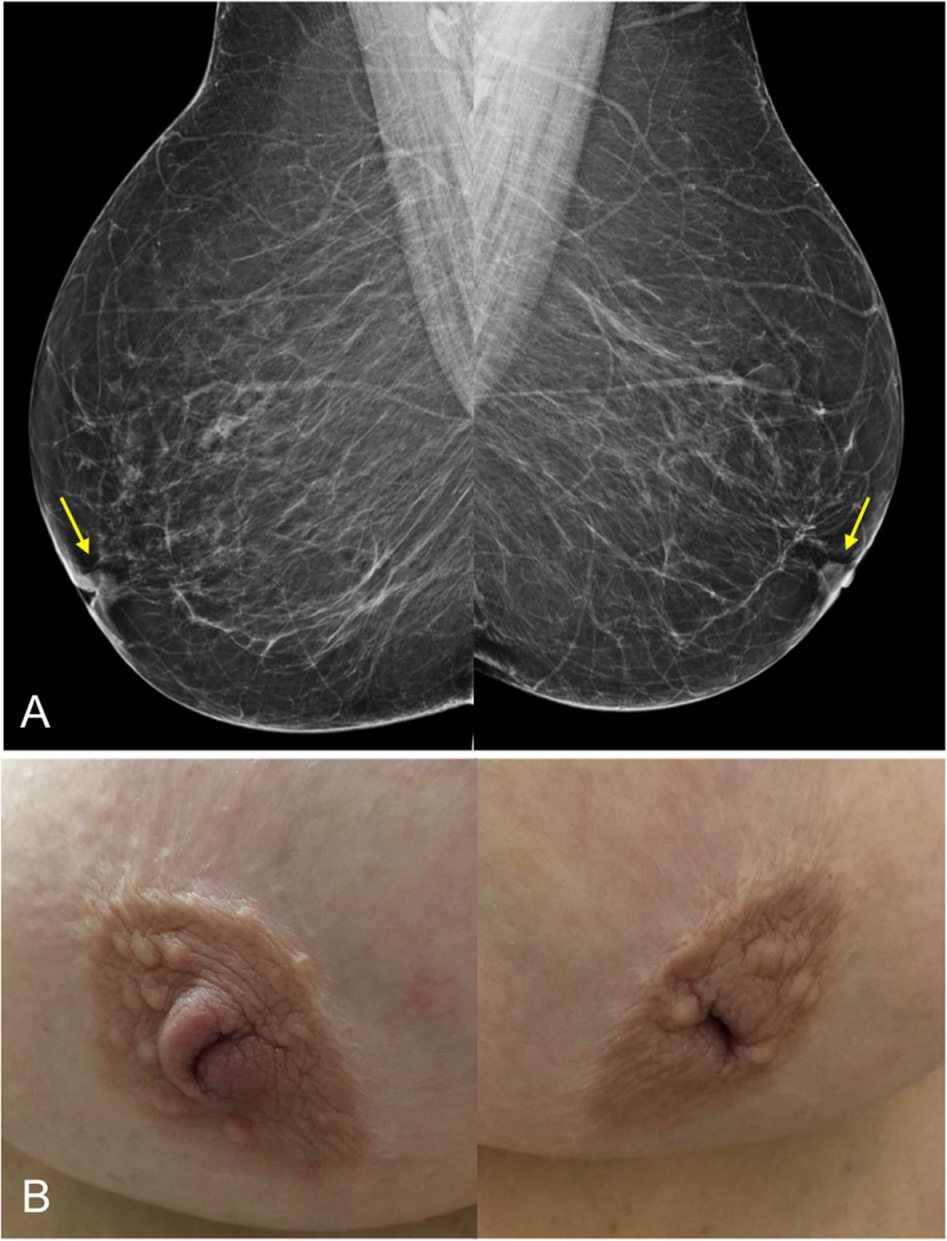
Inverted nipples. A 60-year-old woman with inverted nipples. **a** Craniocaudal 2D mammograms show bilateral inverted nipples that are perfectly tangential and symmetrical. **b** Photograph of the same patient

It is essential to position the breast correctly when acquiring a mammogram [23–25]. It is crucial for the nipple to be tangential in at least one projection, and ideally in both the craniocaudal and mediolateral oblique projections (Fig. 5) [23]. It is often necessary to obtain additional projections (compression or magnification) to enable better assessment. Depending on the patient’s body type (e.g., voluminous breasts), it might be impossible to include the entire breast while maintaining the nipple tangential. In these cases, it is helpful to obtain an additional projection centered on the anterior region (Fig. 6). In patients with inverted nipples (a normal variant), the nipples should be tangential and symmetrical (Fig. 7).

**Fig. 8:**
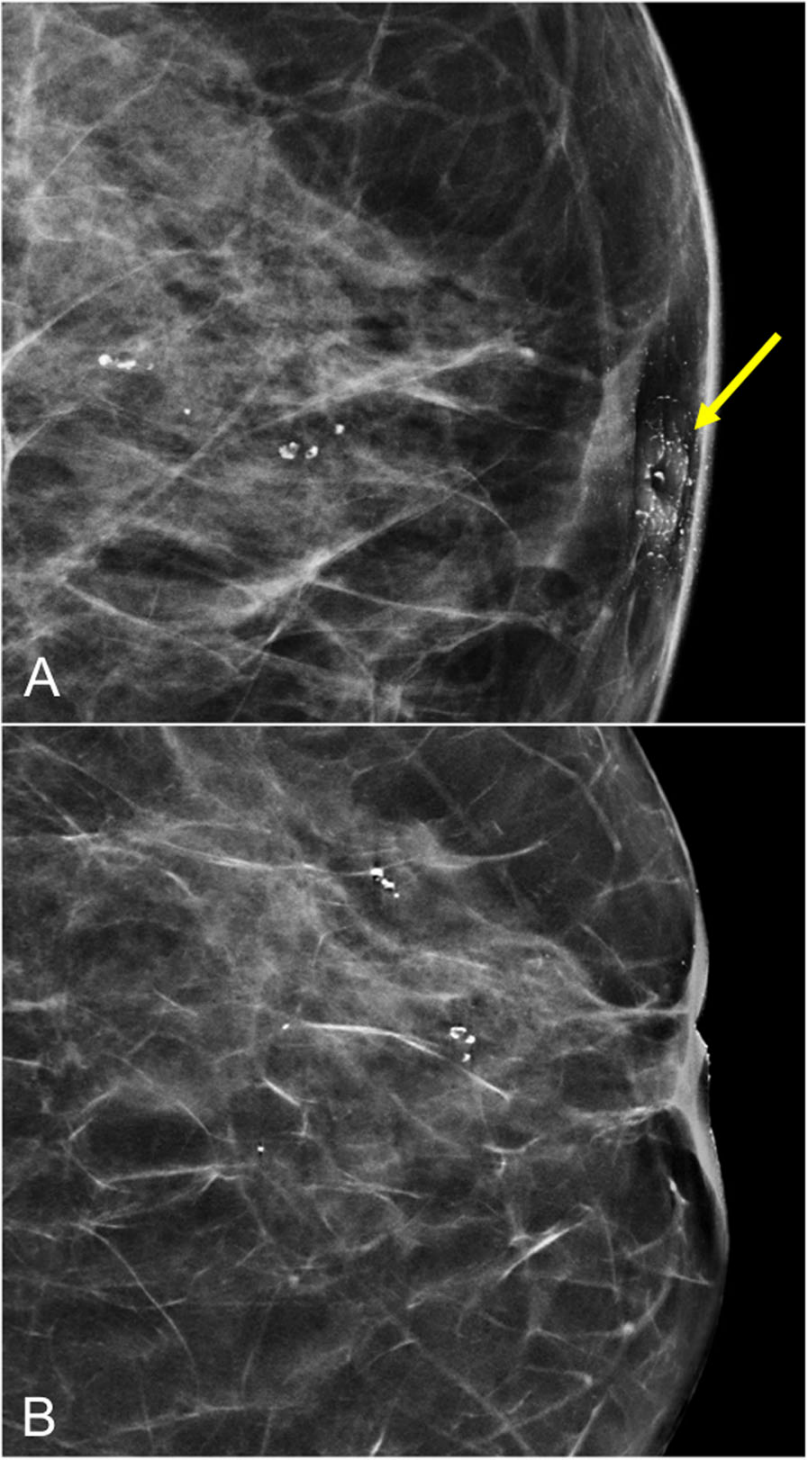
Pitfall. **a** Synthesized craniocaudal 2D mammogram shows multiple dense punctiform images at the level of the nipple mimicking calcifications; these artifacts were caused by remnants of body cream. **b** Repeat image after cleansing the nipple shows the artifacts have disappeared

To avoid pitfalls, it is important to eliminate traces of creams or talcum powder from the skin of the nipple (Fig. 8). Skin lesions should be marked to avoid false-positives. Digital tomosynthesis can help reduce superposition artifacts and differentiate between skin lesions and intramammary lesions [26, 27].

**Fig. 9:**
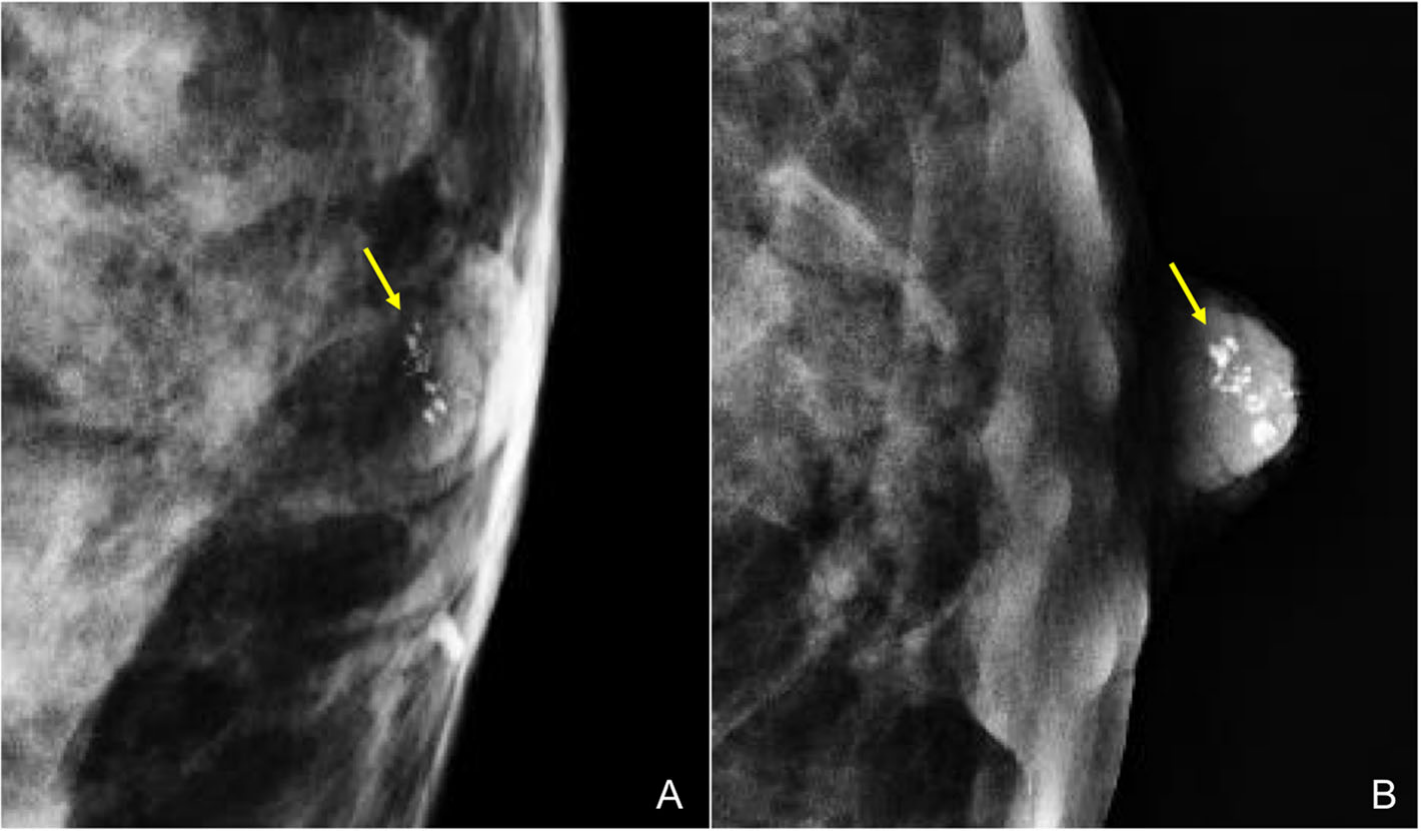
Nipple calcifications. Screening mammogram in a 54-year-old woman. **a** Craniocaudal view of the left breast shows a group of calcifications in the retroareolar region (note that the nipple is not tangential). **b** Magnified view with the nipple perfectly tangential, confirming that the calcifications have benign characteristics and are located in the nipple

**Fig. 10:**
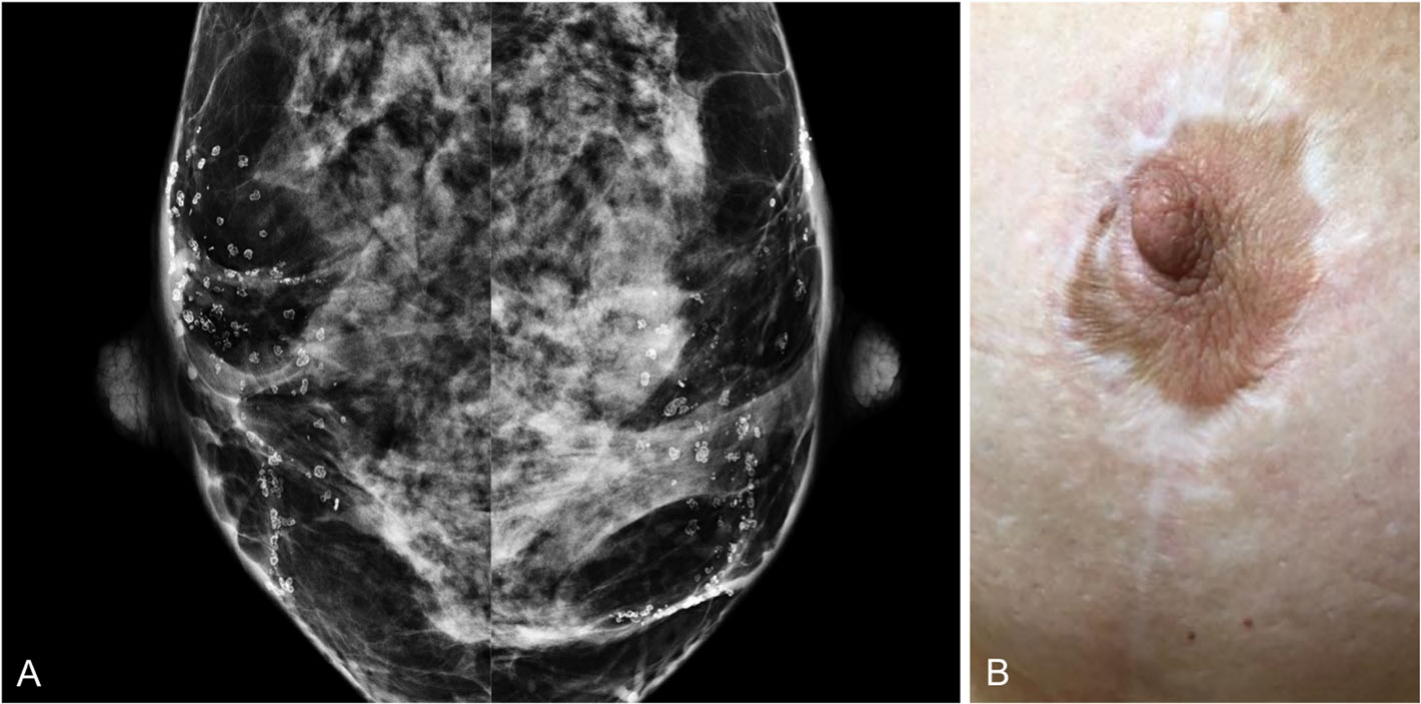
Periareolar calcifications. A 47-year-old woman with a history of breast reduction surgery. **a** Synthesized craniocaudal 2D mammogram shows bilateral periareolar calcifications. **b** Photograph shows the periareolar scar

Mammography is the most sensitive technique for detecting calcifications [27]. In the nipple-areolar complex, calcifications are uncommon and usually benign (cutaneous, secondary to mastoplasty, calcified intraductal detritus, calcifications due to fat necrosis, etc.) (Figs. 9 and 10) [28]. Microcalcifications can also be seen in relation to intraductal carcinoma, sometimes associated with Paget’s disease [29].

### Ultrasound

Ultrasound is very useful in the study of the nipple-areolar complex. In addition to being widely available and not requiring ionizing radiation, ultrasound provides good spatial resolution of this superficial region, making it possible to characterize small lesions in the retroareolar region (especially in dense breasts). Ultrasound is also used to guide percutaneous biopsies [22].

It is advisable to use abundant gel or even a standoff pad to avoid air bubbles between the probe, the skin, and the nipple that can otherwise cause a posterior acoustic shadow [4].

**Fig. 11:**
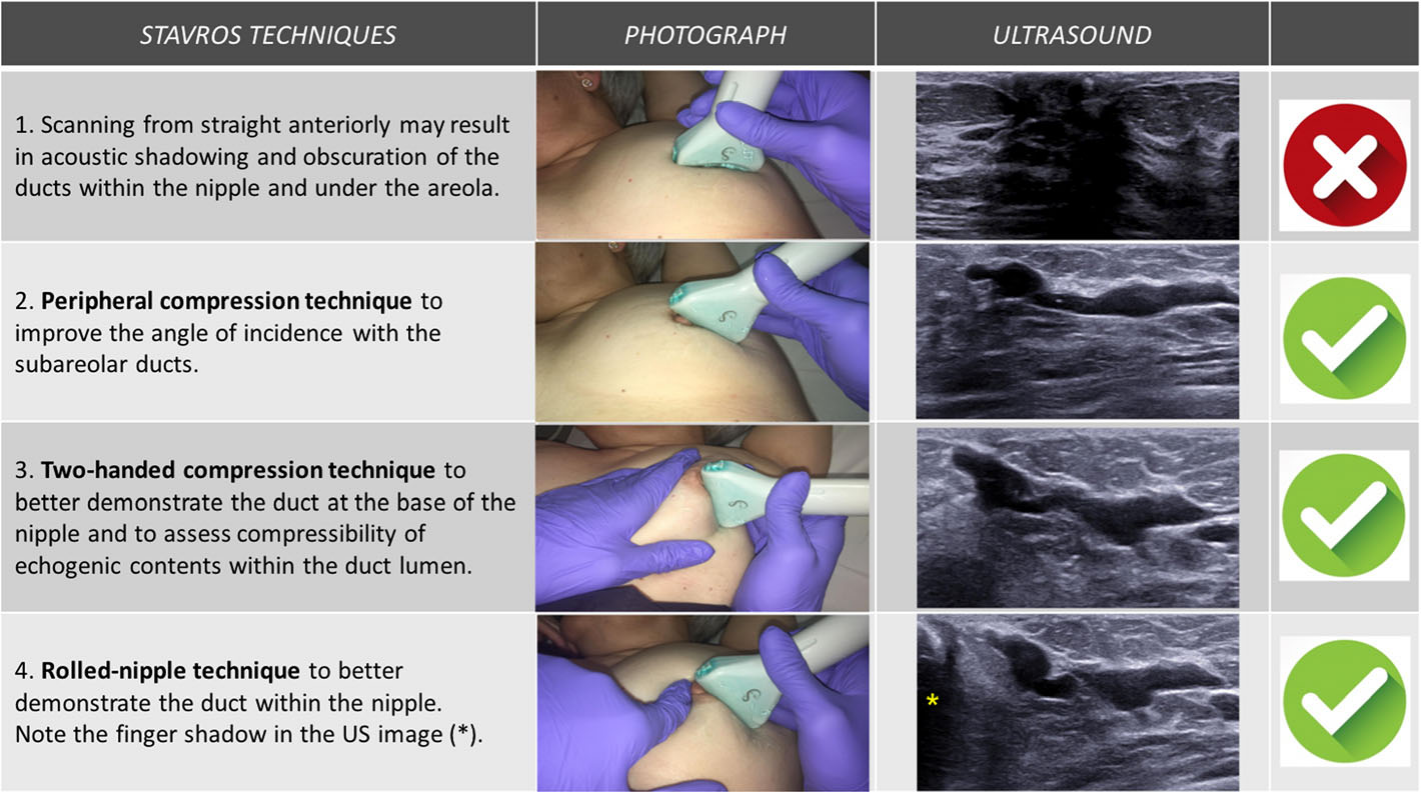
US techniques. Stavros ultrasound techniques to best demonstrate the subareolar and intranipple ducts

It is helpful to angle the probe radially so that the ultrasound beam hits the major axis of the duct perpendicularly to enable the entire length of the duct to be seen [30]. Stavros et al. [30] described various techniques for evaluating the nipple-areolar complex with ultrasound (Fig. 11). Of these, peripheral compression with the probe itself is the one that achieves the best angle of incidence on the subareolar ducts. The two-handed compression technique enables better assessment of the duct at the base of the nipple and also makes it possible to differentiate between an intraductal mass and secretions by checking the compressibility of the echogenic contents of the duct: ducts containing debris collapse with external compression, whereas those containing masses do not [22, 30]. Finally, the “rolled-nipple technique” is useful for evaluating the duct within the nipple [22, 30].

### Magnetic resonance imaging

**Fig. 12:**
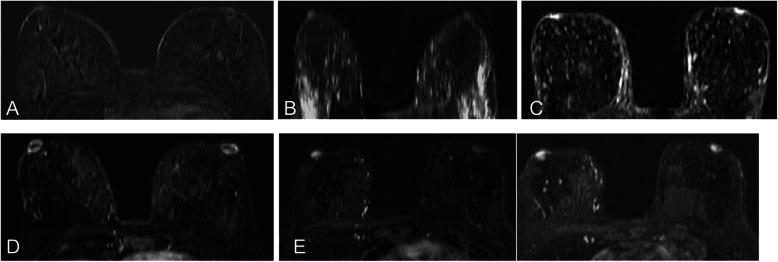
Physiological enhancement in the nipple-areolar complex. Axial contrast-enhanced T1-weighted spoiled gradient-echo (subtracted) images show various degrees of enhancement in a normal nipple, including none (**a**), mild symmetric enhancement (**b**), intense symmetric enhancement (**c**), a thin symmetric ring of enhancement (**d**), and asymmetric early enhancement with symmetric late enhancement (**e**)

Physiological uptake of contrast material in the nipple-areolar complex can manifest in different ways (Fig. 12). A thin symmetrical ring of enhancement is usually seen in both breasts; sometimes enhancement is asymmetrical in the early phase, becoming symmetrical in later phases [31]. In a recent study of 530 normal nipples in 265 asymptomatic women, Gao et al. [32] described three areas of enhancement in subtracted T1-weighted images of the nipple-areolar complex acquired on a 3T scanner and their correlation with pathology findings: (a) superficial linear enhancement (SLE); (b) nonenhancing zone (NEZ); (c) internal nipple enhancement (INE).

The nipple is everted in 75% of women, flat in 23%, and inverted in 2% [32]. MIP images are very useful for assessing the morphology and symmetry of the nipple-areolar complex. On postcontrast images, the nipple should be hypointense or isointense to the enhanced parenchymal tissue in the background [32].

**Fig. 13:**
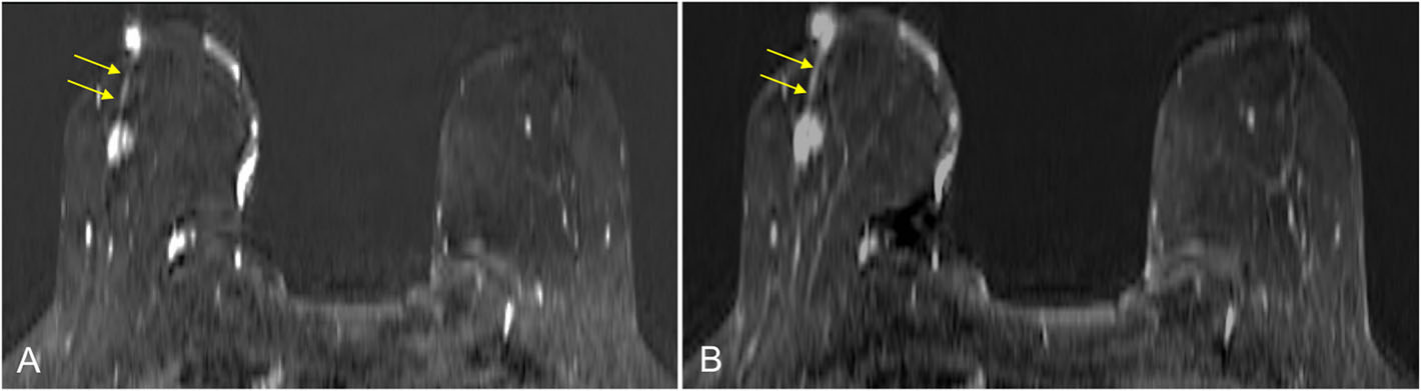
Pathological enhancement in the nipple-areolar complex. A 71-year-old woman. Axial contrast-enhanced T1-weighted spoiled gradient-echo (subtracted) images show asymmetric irregular nodular early enhancement (**a**) that is maintained in late phases (**b**) secondary to involvement by invasive ductal carcinoma. Irregular-shaped masslike enhancement in the middle third of the junction of the outer quadrants in the right breast with linear uptake and segmental distribution to the nipple-areolar complex, compatible with an intraductal component (arrows)

Preoperative assessment of nipple-areolar complex tumor involvement is essential for staging (prognosis) and therapeutic management [33]. In the evaluation of tumor involvement of the nipple-areolar complex, MRI has high sensitivity (90–100%), moderate specificity (80–90%), and a high negative-predictive value (98%) [6, 7, 34], being especially useful in cases with uncertain findings on conventional imaging tests, even in the absence of clinical suspicion [5, 35]. MRI’s usefulness derives from its better soft-tissue resolution and the information from dynamic contrast enhancement. Asymmetrical, nodular, irregular, early, or persistent enhancement should raise suspicion of malignancy (Fig. 13) [6, 28]. Moreover, as nipple-sparing mastectomy is becoming more common for cancer treatment or for prophylaxis in high-risk patients [36, 37], preoperative determination of the tumor-to-nipple distance is fundamental to ensure safety [38, 39].

Finally, MRI is also useful to complement mammography and ultrasound in the diagnostic management of pathological nipple discharge and to guide percutaneous biopsy [40–44].

### Galactography

Galactography is indicated in patients with pathological discharge from the nipple (unilateral, from a single duct, spontaneous, and persistent with a clear, serous, or bloody appearance) and negative findings on mammography and ultrasound [45].

**Fig. 14:**
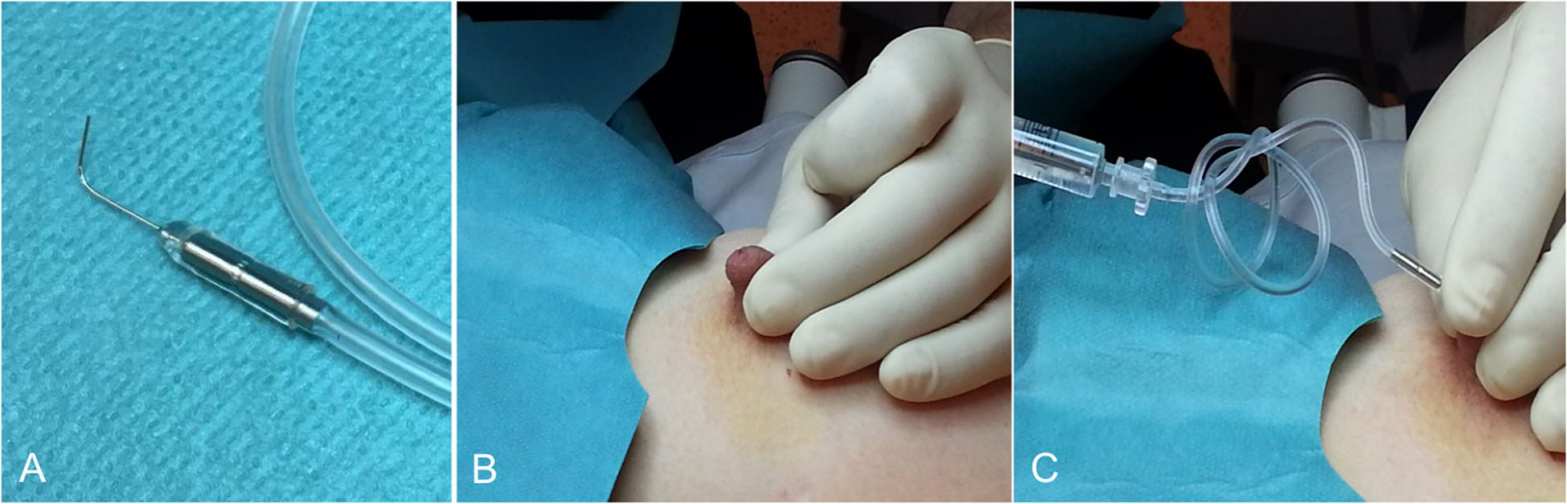
Galactography technique. 30-gauge cannula (**a**); the nipple must be firmly stabilized between the thumb and forefinger (**b**); canalization of the discharging orifice and contrast injection (**c**). A magnified craniocaudal view is obtained with the cannula taped in place and the breast compressed

**Fig. 15:**
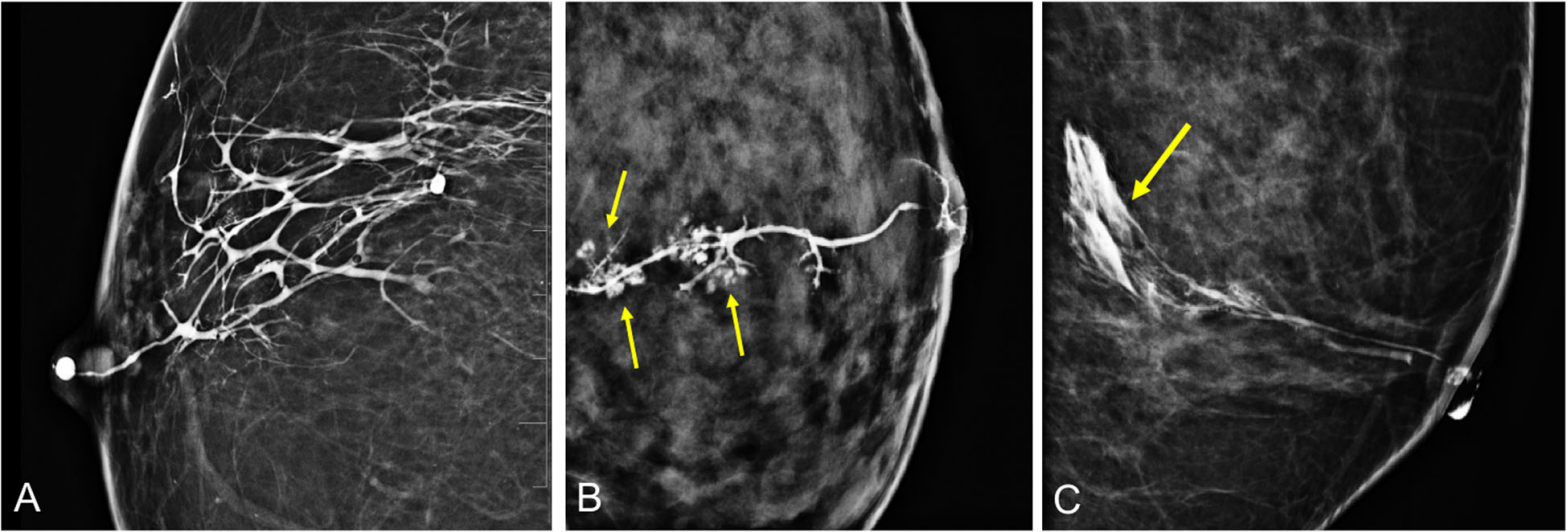
Ductograms. **a** Normal ductogram, craniocaudal view. Note the normal “lobular blush” in (**b**) (arrows), caused by the contrast material filling the lobular portion of the terminal ductal lobular unit. Ninety-degree mediolateral ductogram (**c**) shows delayed extravasation from excess injection pressure (arrow)

To canalize the secreting orifice and inject undiluted iodinated contrast material, the nipple must be gently but firmly secured between the thumb and forefinger. A 30-gauge cannula is used (Fig. 14). With the cannula fixed in place, a magnified craniocaudal projection is obtained [45]. Patients may experience local pain if contrast material extravasates (Fig. 15) [45].

**Fig. 16:**
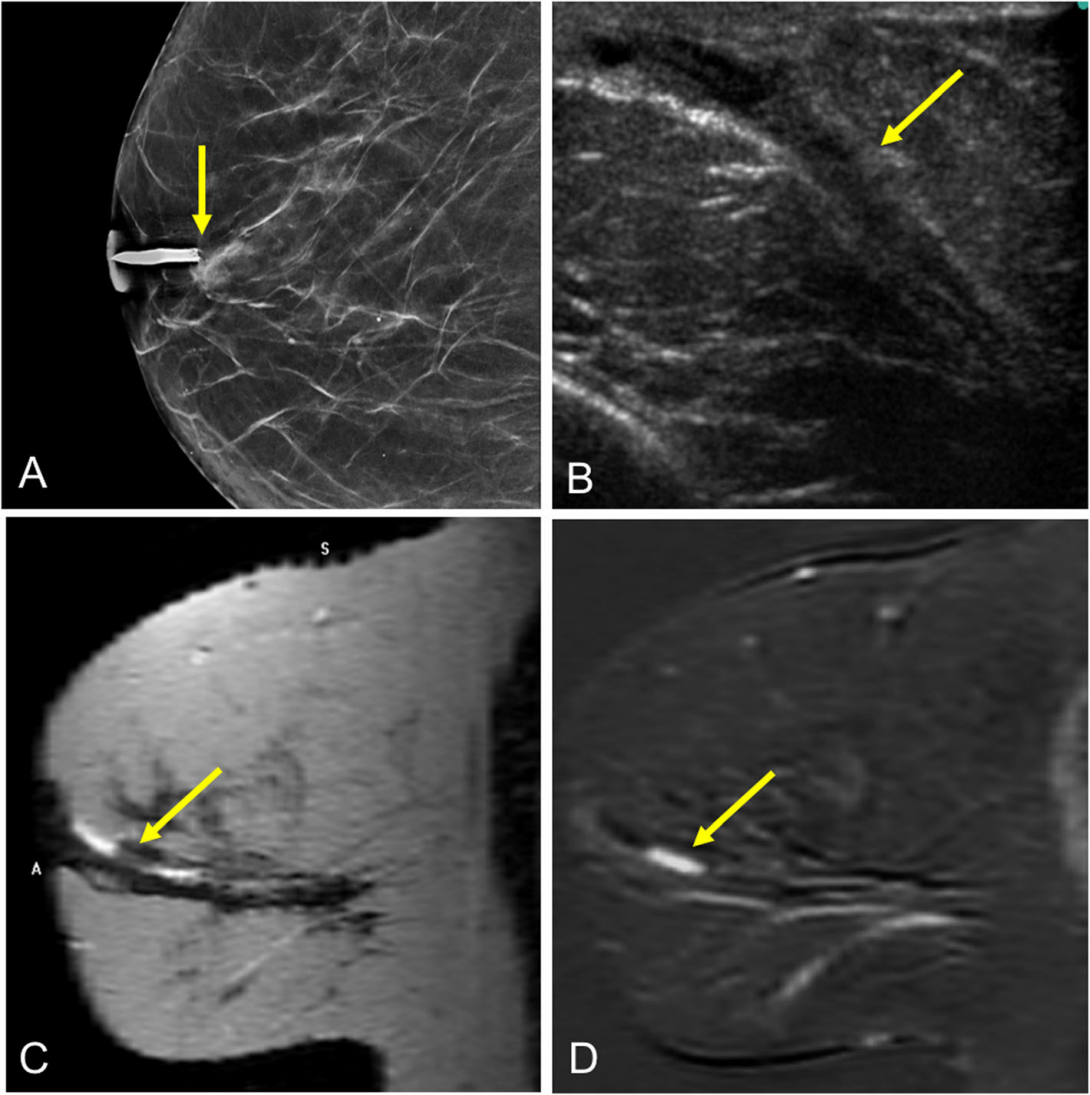
Ductal cutoff on galactography. A 65-year-old woman with spontaneous bloody discharge from a single orifice in the right breast and negative findings at mammography and ultrasound (images not shown). **a** Craniocaudal ductogram shows a concave filling defect situated 2 cm behind the nipple. **b** Ultrasound obtained after galactography shows ductal ectasia with an intraductal lesion (arrow). **c** Sagittal T2-weighted MRI shows hyperintense ductal ectasia with an intraductal mass, which on (**d**) sagittal contrast-enhanced T1-weighted MRI (subtracted image obtained 120 s after contrast injection) corresponds to a mass with differential contrast enhancement (arrow). Histologic study: solitary intraductal papilloma

**Fig. 17:**
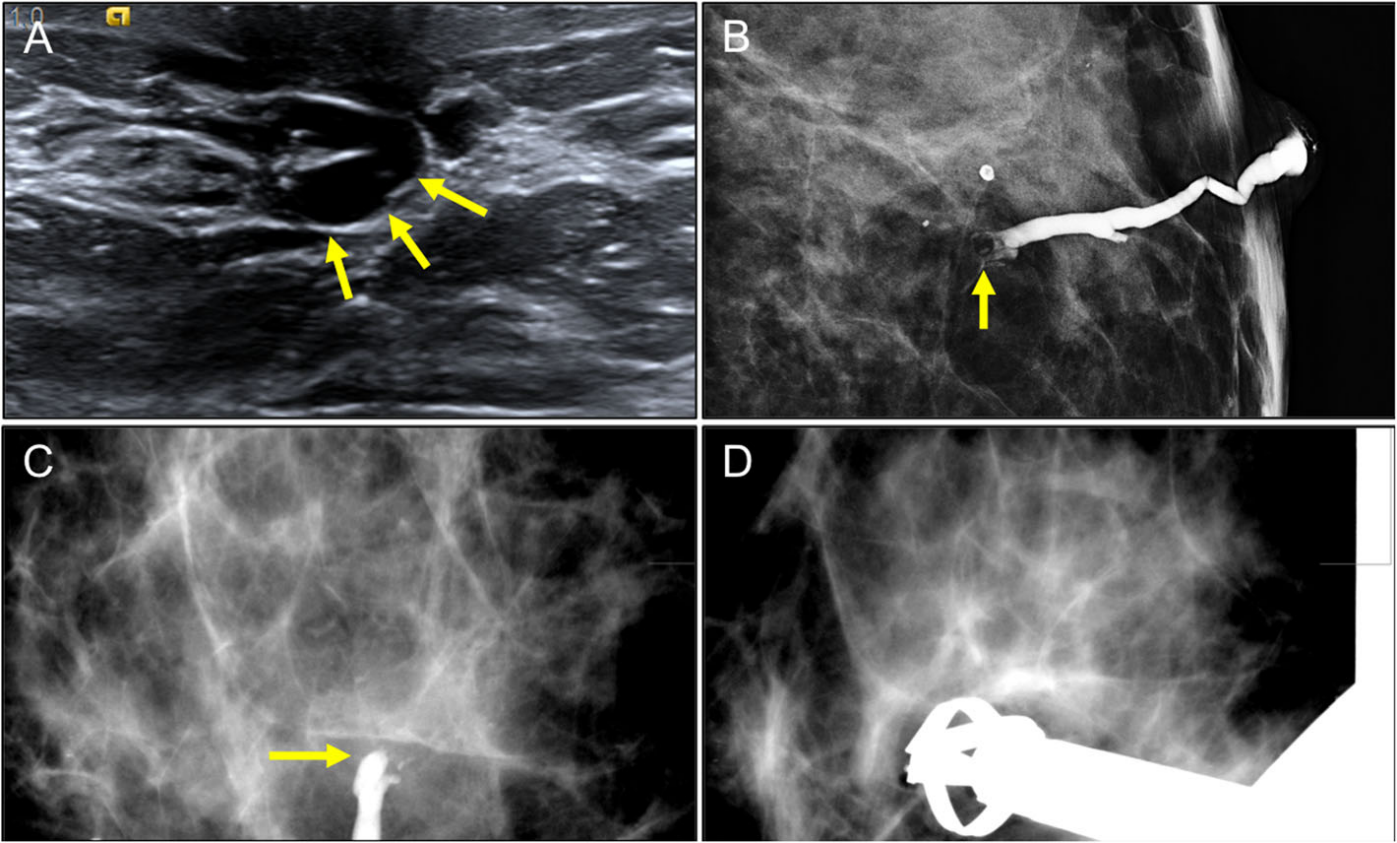
Galactography-guided percutaneous excision biopsy. A 59-year-old woman with a 1-week history of serous discharge from a single orifice in her right nipple. **a** Ultrasound shows retroareolar ductal ectasia without apparent intraductal lesions (arrows). **b** Craniocaudal ductogram shows a concave cutoff situated 2.5 cm behind the nipple. **c** Image of the lesion at the level of the cutoff obtained with the patient positioned prone on the stereotactic table. **d** The lesion was excised with the Intact-BLES™ biopsy system (Medtronic Inc., Dublin, Ireland). Histology diagnosed intraductal papilloma

The main aim of galactography is to detect intraductal disease and to locate the pathological duct, and this information is useful for planning surgery [46]. However, with the development of MRI techniques, the indication for galactography has become controversial [40, 47]. Although some authors do not include galactography in the diagnostic algorithm for the radiological management of nipple discharge [22, 40–42], recent studies reaffirm its usefulness, given its high sensitivity and high negative predictive value when combined with mammography and ultrasound (Fig. 16) [47–50]. Finally, galactography can be used to guide interventional procedures for intraductal lesions (Fig. 17) [51].

An alternative to conventional galactography is MR galactography, which has the advantage of showing the duct and the full extent of potential underlying disease [52].

## Benign disease

### Duct ectasia

**Fig. 18:**
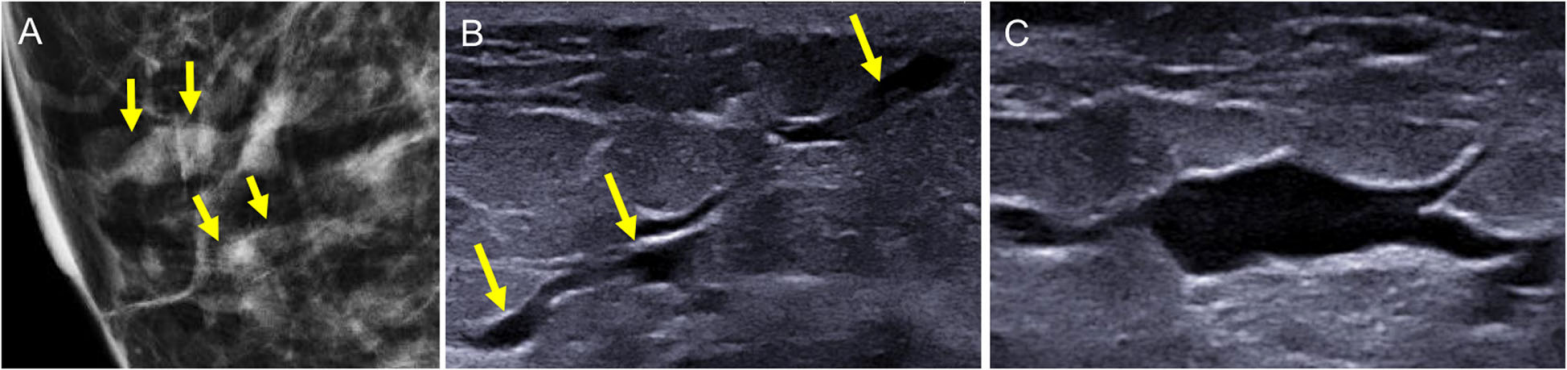
Ductal ectasia. Different examples of ductal ectasia without interior contents seen on mammography (**a**) and ultrasound (**b, c**)

First described by Haagensen [53], mammary duct ectasia is a benign process characterized histologically by dilated ducts, variable degrees of periductal inflammation, and progressive fibrosis [11]. Ductal ectasia can occur at any age, although it is most common after 50 years of age [54]. Ectasia predominantly affects the ducts in the retroareolar region, bilaterally and symmetrically. Patients may be asymptomatic (most commonly) or have nipple retraction, secretion, or a palpable subareolar nodule [28]. By definition, the duct measures greater than 2 mm in diameter and greater than 3 mm in the ampullary portion [8, 13]. Duct ectasia can be visible on mammograms, especially in predominantly fatty breasts. It manifests as radiodense tubular structures that converge in the nipple-areolar complex (Fig. 18). The presence of benign-appearing calcifications in the dilated subareolar ducts is a common mammographic finding [8].

**Fig. 19:**
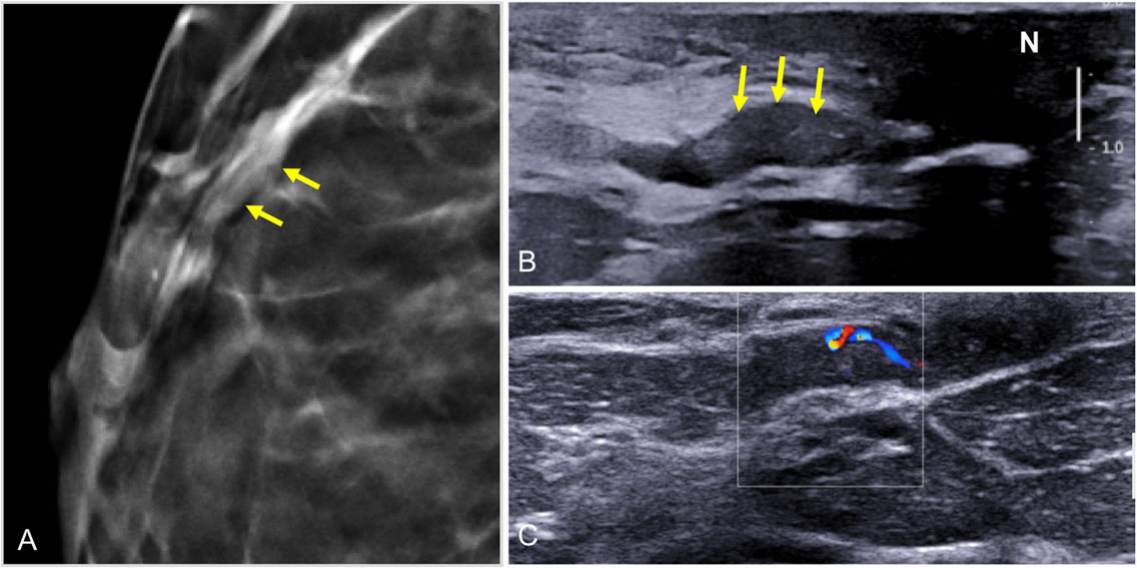
Ductal ectasia with intraductal contents. Solitary intraductal papilloma. A 47-year-old woman with serous secretion from a single orifice in the right nipple. **a** Craniocaudal tomosynthesis slice shows a nodular image with well-defined borders in the retroareolar region of the outer quadrants with a segmental distribution (arrows). **b** Ultrasound shows ductal ectasia with a well-defined solid nodular lesion (arrows) adjacent to the nipple (N). **c** Doppler signal due to flow inside the intraductal lesion

On ultrasound, duct ectasia is seen as single or multiple tubular structures that can be anechoic or have echoes inside due to debris or infection. Ultrasound can differentiate between debris and an intraductal mass. Apart from compressing the duct to check to see whether it collapses, Doppler studies can be very useful because intraductal masses can have flow signals inside them that indicate vascularization [8]. It is important to remember that duct ectasia associated with an intraductal lesion or other suspicious sign should be biopsied (Fig. 19). The ultrasound characteristics that should raise suspicion of an underlying malignant process are peripherally located duct ectasia (outside the retroareolar region), diffuse irregularity of the margins of the duct, focal wall thickening, and a hypoechoic lesion adjacent to the duct, as well as asymmetrical duct ectasia [55, 56].

**Fig. 20:**
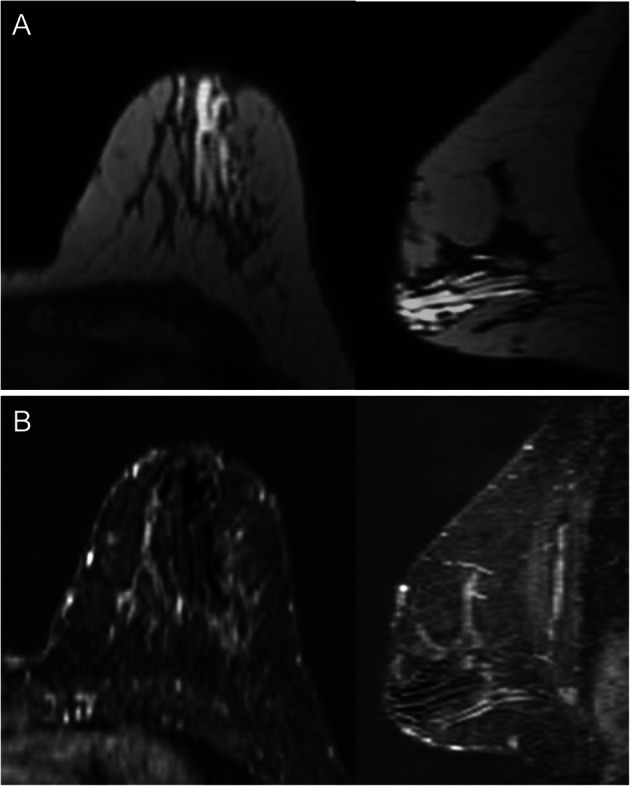
Ectasia and MRI. Screening MRI in an asymptomatic high-risk 38-year woman 6 months after lactation. **a** Unenhanced axial and sagittal T1-weighted images. **b** Contrast-enhanced axial and sagittal T1-weighted MRI (subtracted image obtained 120 s after contrast injection). Note the tubular structures in the retroareolar region of the left breast with a segmental distribution; proteinaceous material causes increased signal intensity on T1-weighted sequences, but not intraductal enhancement

On MRI, duct ectasia is usually seen as tubular structures with a segmental distribution that have high signal intensity on T2-weighted sequences and variable signal intensity on T1-weighted sequences, depending on the proteinaceous and/or hematic contents (Fig. 20) [57]. If the ducts are not pathologic, they do not enhance after the administration of contrast material. In some cases with intraductal inflammation or disease, rounded, smooth-margined ring enhancement, or even heterogeneous non-mass-type enhancement is seen [57].

### Periductal mastitis

Periductal mastitis is a suppurative inflammatory process that occurs mainly in non-lactating premenopausal women [58]. It is characterized by periductal inflammation with an infiltrate consisting predominantly of plasma cells [59]. Its etiology is uncertain, although it could be related to bacterial infection and obstruction of the subareolar ducts [60]. Risk factors include smoking, obesity, and diabetes [60].

**Fig. 21:**
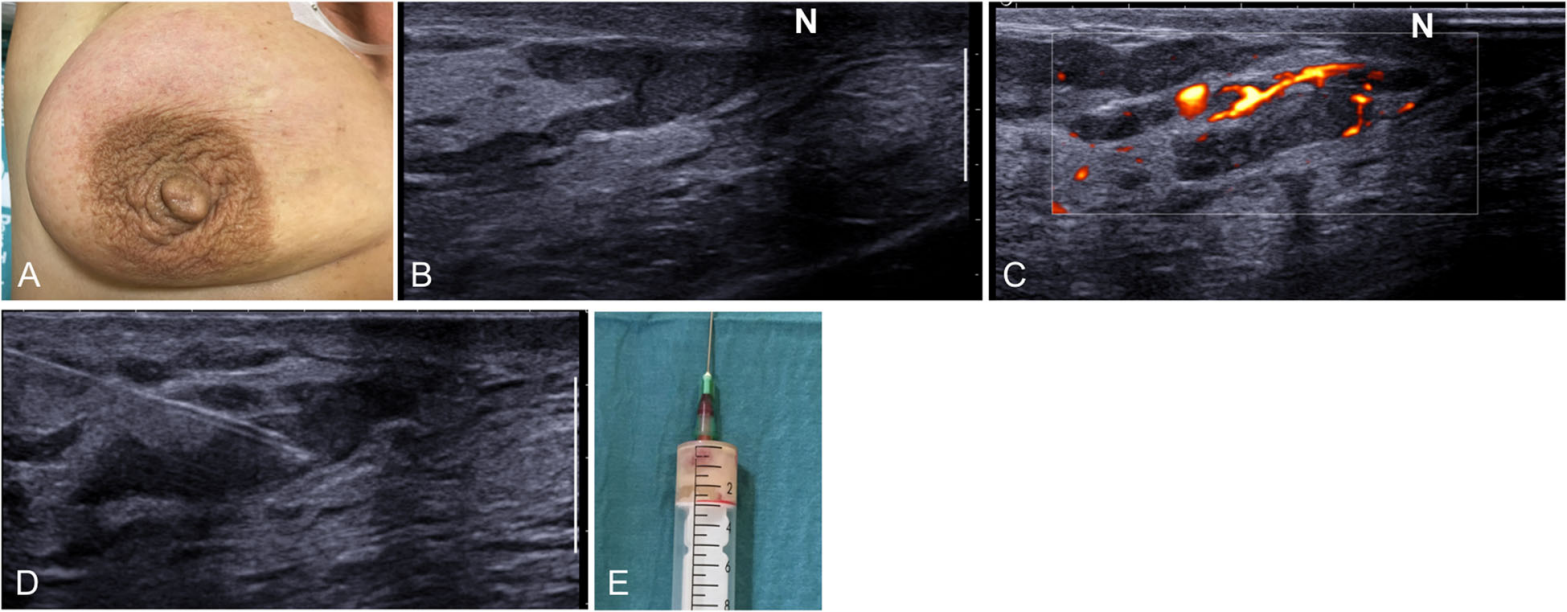
Periductal mastitis. Photograph of a 59-year-old smoker with a painful erythematous area in the upper outer quadrant of her right breast (**a**). Ultrasound shows skin thickening and retroareolar ductal ectasia with echogenic contents (**b**) and increased periductal Doppler signal (**c**). Fine-needle aspiration obtained purulent material (pus) (**d**, **e**)

**Fig. 22:**
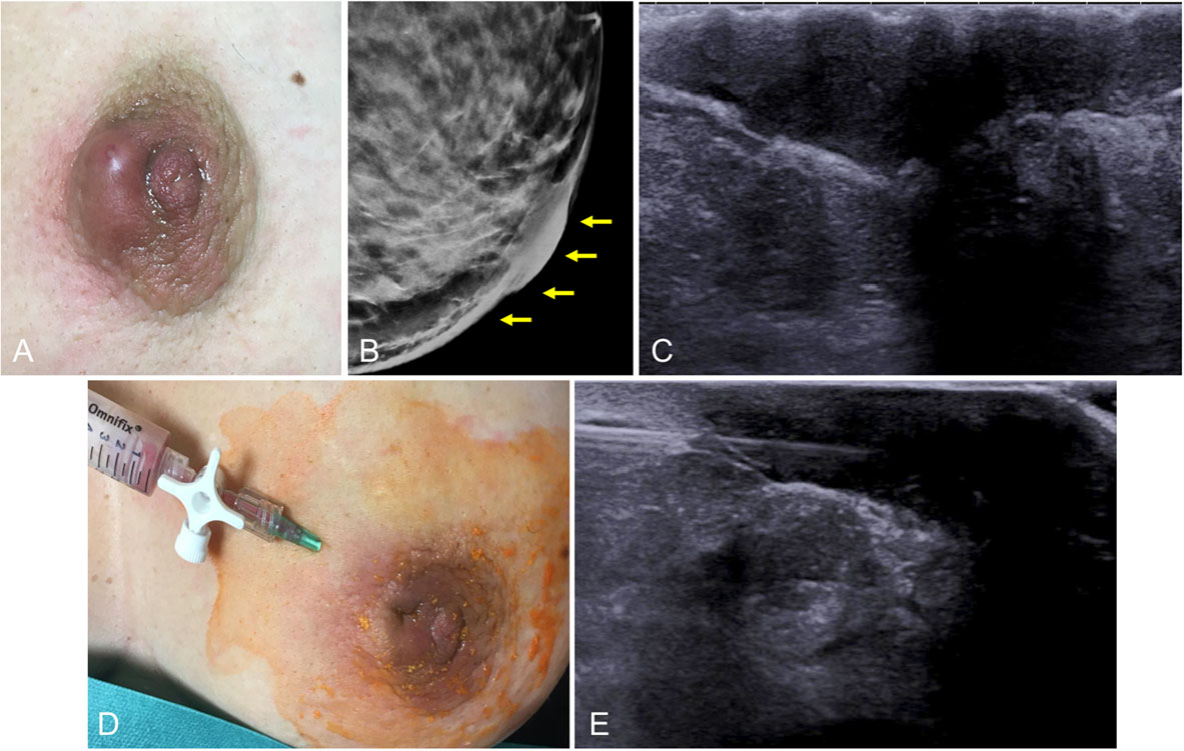
Subareolar abscess. Photograph of a 42-year-old woman with a painful erythematous palpable areolar mass in her left breast with mild involvement of the adjacent skin (**a**). Synthesized 2D mammogram shows marked skin thickening of the nipple-areolar complex without other underlying findings (**b**). Ultrasound shows a heterogeneous hypoechogenic intradermal collection compatible with an abscess (**c**). Fine-needle aspiration was able to drain the collection completely (purulent material), and the patient was prescribed antibiotics and follow-up (**d**, **e**)

The clinical presentation consists of reddening and pain in the areola that may be accompanied by nipple discharge or inversion [8]. Ultrasound shows duct ectasia with intraductal pus and increased periductal Doppler signal (Fig. 21). On rare occasions, periductal mastitis is associated with retroareolar abscesses, which appear as heterogeneous ill-defined hypoechoic masses with a posterior acoustic shadow (Fig. 22), requiring a differential diagnosis with carcinoma. Thus, the clinical context and the response to antibiotics are key for the diagnosis. In case of doubt, clinical and ultrasound follow-up at four to 6 weeks is recommendable [61].

### Zuska’s disease

**Fig. 23:**
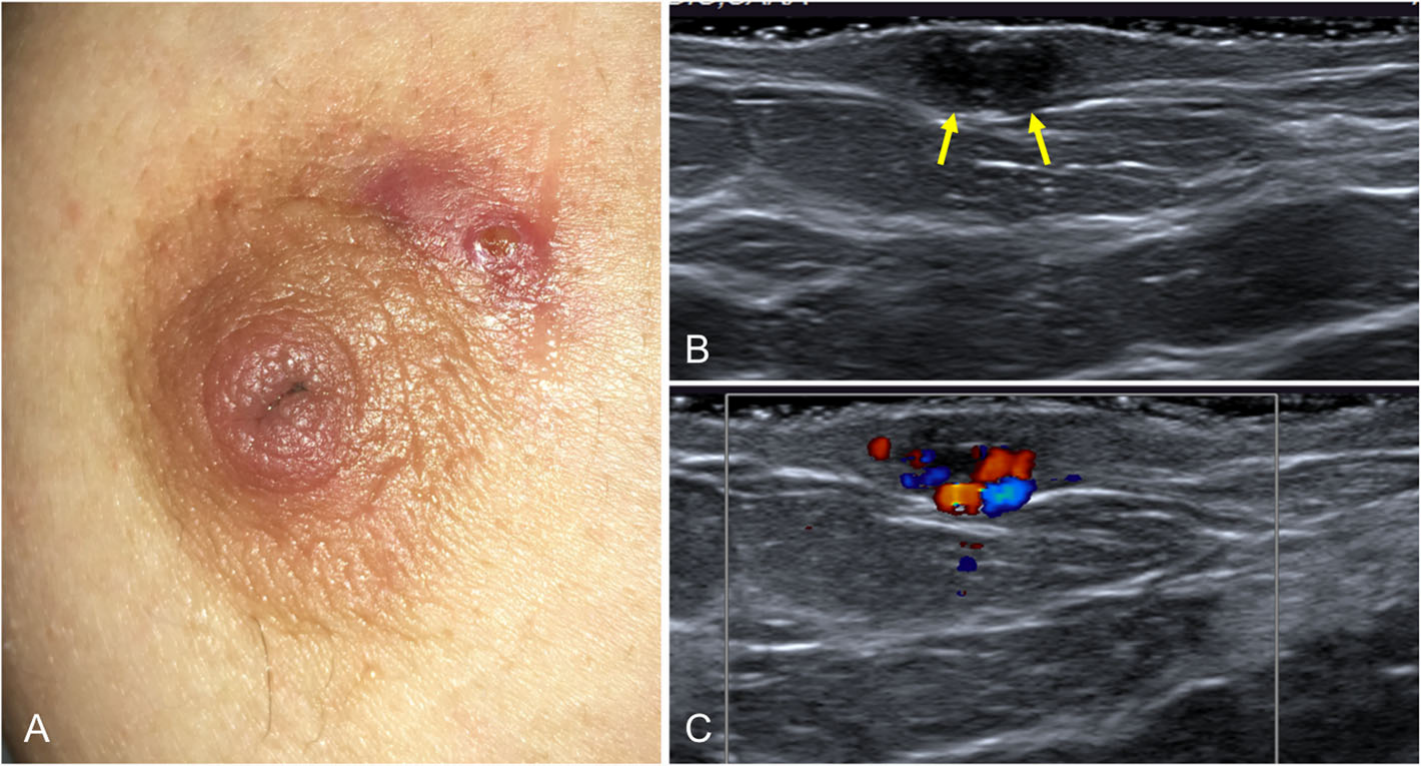
Zuska’s disease. **a** Photograph of a 47-year-old woman with a history of smoking who had a recurrent painful erythematous palpable subareolar mass with a small secreting ulcerous lesion. **b** Ultrasound shows skin thickening with a small hypoechogenic collection below the lesion (arrows), with increased peripheral color-Doppler signal (**c**)

Zuska first described this recurring periareolar fistula in 1951 [62]. Zuska’s disease consists of the formation of spontaneously draining subareolar abscesses that form chronic periareolar fistulas [62]. It predominantly affects non-lactating middle-aged women [63], and is directly associated with smoking [63, 64]. It presents as a painful, erythematous subareolar mass and recurring fistula at the edge of the areola. Clinical suspicion is key because the definitive treatment is surgical resection of the fistula and the involved duct [65]. The diagnosis is clinical, but ultrasound is useful for assessing the extent of the disease (Fig. 23).

### Benign tumors

The vast majority of masses located in the nipple and subareolar region are benign.

### Solitary intraductal papilloma

Although an intraductal papilloma can appear at any age, it is most common between the third and fifth decades of life, and has a low incidence in men [66, 67]. Intraductal papillomas are proliferative lesions in which a vascular-connective axis is lined with a layer of myoepithelial or basal cells and another layer of luminal epithelial cells [11]. Intraductal papillomas originate in the proximal or retroareolar ducts and generally cause obstruction and ectasia of the duct [68]. Intraductal papilloma is the most common cause of pathological nipple discharge (48%), followed by duct ectasia (15–20%) [69]. Less frequently, this lesion can present as a palpable mass [66].

**Fig. 24:**
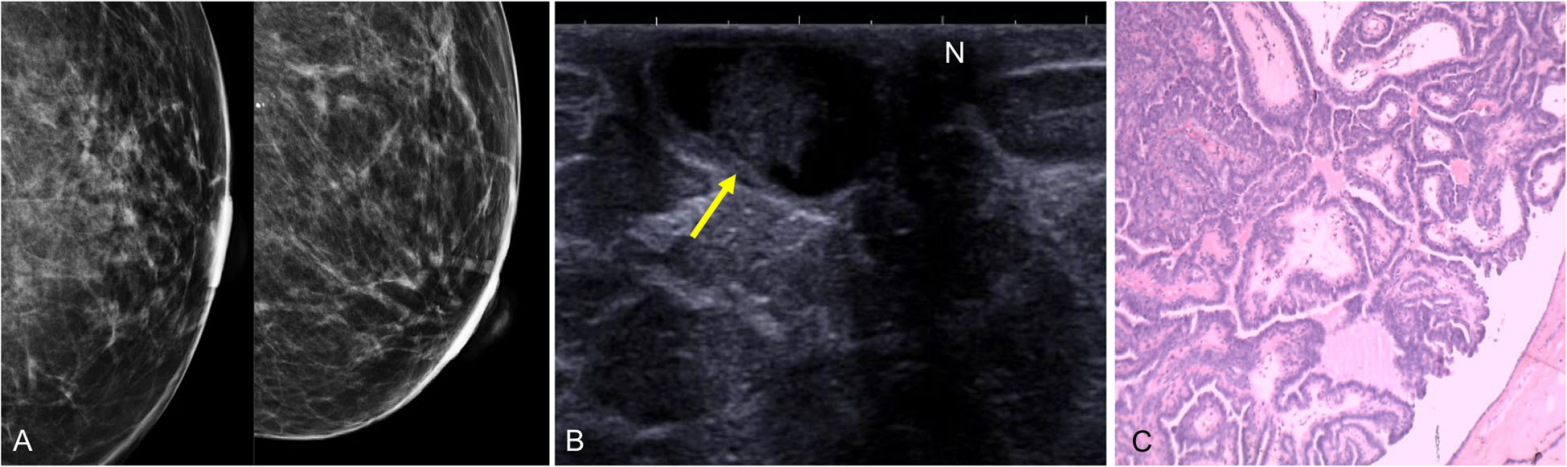
Solitary intraductal papilloma. A 68-year-old woman with a palpable retroareolar nodule in her left breast. **a** Normal findings on craniocaudal and mediolateral oblique 2D mammograms. **b** Ultrasound shows a cystic lesion with a solid nodule inside it (arrow) adjacent to the nipple (N). **c** Hematoxylin-eosin stain (× 4) shows branching intraductal structures consisting of a central fibrovascular axis surrounded by epithelial and myoepithelial cells

**Fig. 25:**
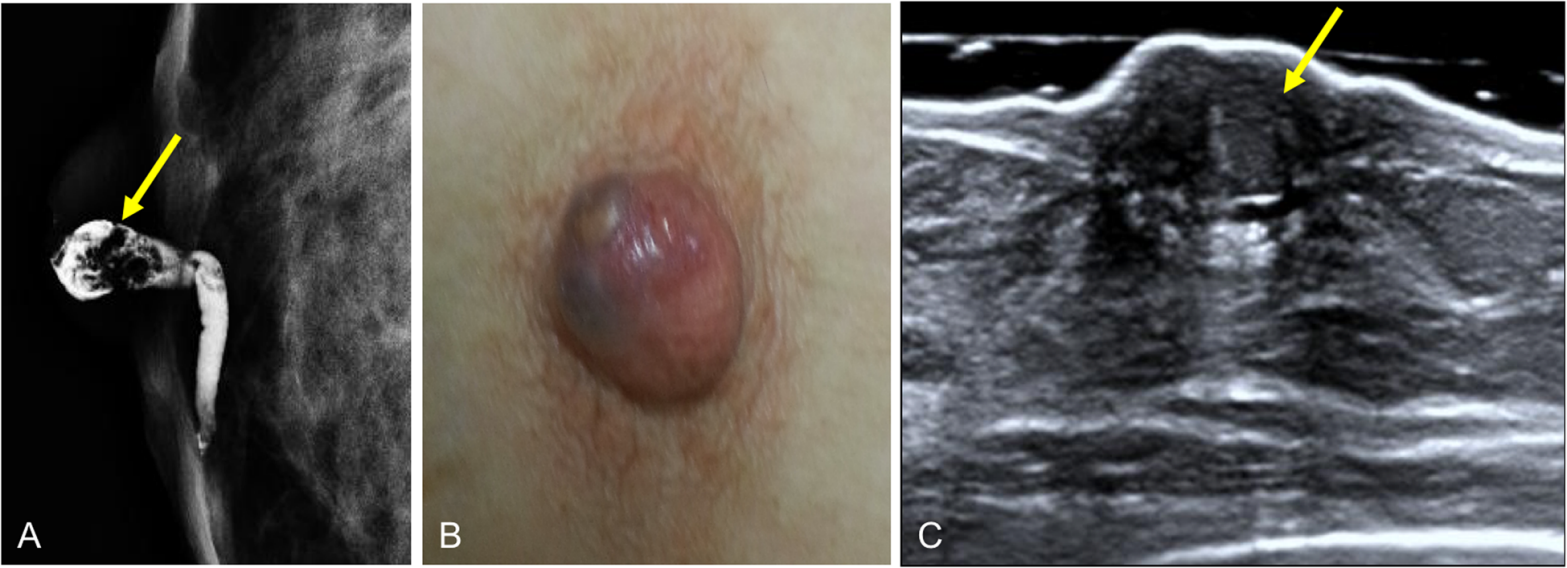
Intraductal filling defect. A 48-year-old woman with bloody discharge from a single orifice in the right breast and negative findings at mammography and ultrasound (images not shown). **a** Craniocaudal ductogram shows a filling defect just behind the secreting orifice (arrow). Histologic study: solitary intraductal papilloma. **b** Photograph of a bluish nodule that appeared in the same breast five years later; **c** ultrasound shows the lesion as a solid nodule (arrow). Histologic study: solitary intraductal papilloma (inverted)

Mammography shows a nodular or oval lesion with well-defined margins in the retroareolar region. Up to 25% have benign calcifications [70]. Small papillomas in the retroareolar region are often occult because of the high density and technical difficulty of evaluating this region by mammography [70]. On ultrasound, they can be seen as an intraductal mass near the nipple (with or without associated ectasia), as an intracystic mass (Fig. 24), or as a predominantly solid lesion that fills the entire duct [61]. Internal flow related to a vascular pedicle may be seen on Doppler imaging (Fig. 19) [70]. Galactography generally reveals an intraductal filling defect (Fig. 25) or ductal cutoff (Fig. 16). On MRI, intraductal papilloma can be seen as an enhancing mass with circumscribed or irregular margins in association with ductal dilation, as ductal dilation without an intraductal mass, or as a solid-cystic mass [71]. Small papillomas might not be visible on MRI.

The upgrade rate for solitary benign intraductal papilloma (without atypia) diagnosed by core biopsy varies widely among series (0–33% )[72], so surgical excision has been the recommended treatment [73]. However, recent studies have shown that US-guided directional vacuum-assisted removal with clinical and imaging follow-up is viable [72, 74] and that cases with good radio-pathologic concordance without associated risk factors could even be managed with imaging alone [75, 76].

### Nipple adenoma

**Fig. 26:**
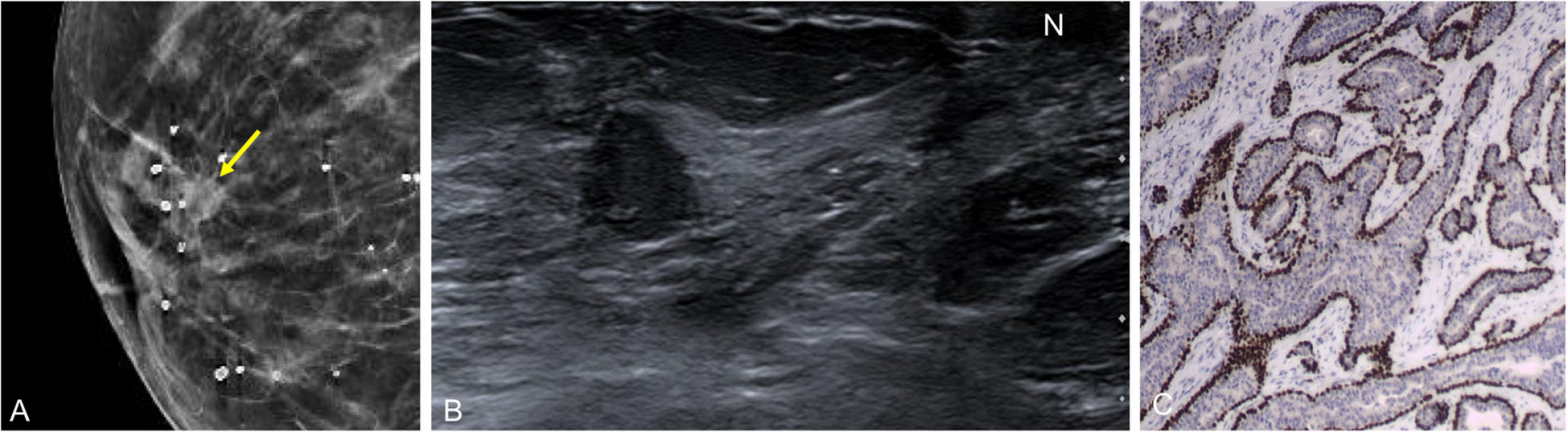
Nipple adenoma. An 81-year-old woman with a several-week history of pain and swelling of the right breast. **a** Mammogram shows an isodense rounded retroareolar mass with slightly irregular margins (arrows); **b** on ultrasound, it is seen as a solid nodular lesion (N: nipple). **c** Immunohistochemistry stain with p63 (× 4) shows glandular and ductal proliferation consisting of epithelial and myoepithelial cells, which express p63

Also known as florid papillomatosis, erosive adenomatosis, and subareolar papillomatosis, nipple adenoma is a rare variant of intraductal papilloma [77]. It originates in the ducts of the nipple and predominantly affects women in the fifth decade of life [12]. Clinically, patients may present with a small palpable nodule below the skin of the nipple, which is typically accompanied by inflammatory changes in the nipple (pain, redness, and swelling). Skin involvement results from the growth of glandular epithelium toward the surface of the skin [12]. The cutaneous symptoms raise suspicion of Paget’s disease, so skin biopsy is usually required for histologic study [78]. Ultrasound shows a hypoechoic nodule in the nipple or subareolar region (Fig. 26). The treatment is surgical; some recent publications describe cryosurgical treatment, a minimally invasive surgical technique, for this benign condition [3].

### Syringomatous tumor of the nipple

**Fig. 27:**
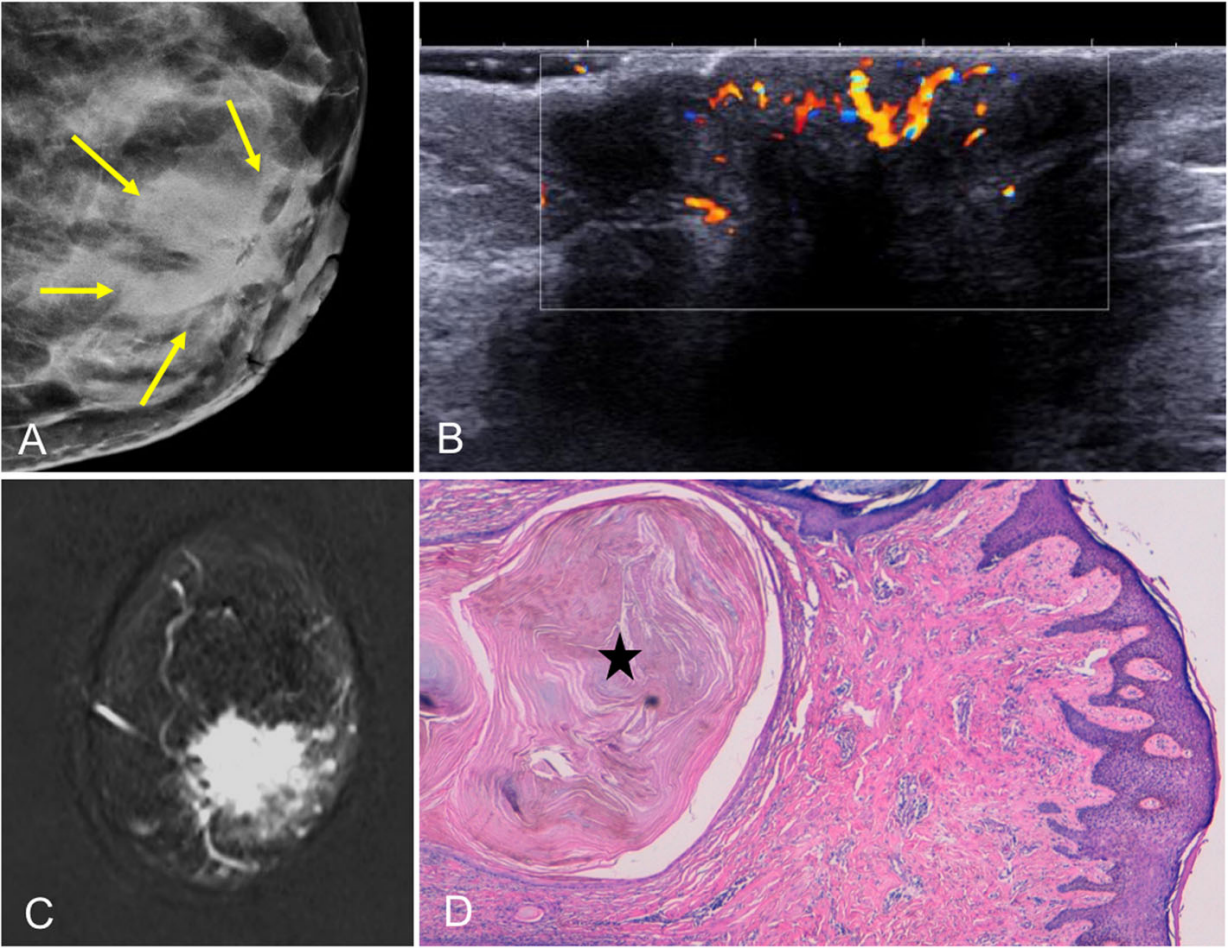
Syringomatous tumor of the nipple. A 47-year-old woman with retraction and hardening of the left nipple. **a** Mammogram shows retraction of the left nipple and asymmetric retroareolar density. **b** On ultrasound, the lesion is solid and hypoechogenic with ill-defined borders and increased peripheral color-Doppler signal. **c** Contrast-enhanced coronal T1-weighted MRI (subtracted image obtained 120 s after contrast injection) shows mass-type uptake with pronounced early enhancement in a lesion with hazy borders that retracts the nipple-areolar complex. **d** Hematoxylin-eosin stain (× 4) shows a proliferation of elongated glandular structures like strings of cells in the dermis, with the formation of keratin cysts (star)

Also called infiltrating syringomatous adenoma [79], this extremely rare benign lesion originates in the eccrine glands of the skin of the nipple and areola [11]. Clinically, it presents as a subareolar nodule (occasionally painful). It can cause nipple discharge or retraction. Rarely, it is associated with ulceration and erosion of the nipple [80]. Although benign, syringomatous adenomas are locally invasive and often mimic a malignant lesion on imaging tests. On mammography, it is usually seen as an irregular hyperdense mass in the subareolar region, although it can also present as pleomorphic calcifications in the nipple [79, 81]. Ultrasound shows an ill-defined subareolar mass with heterogeneous echoes inside (Fig. 27).

The diagnosis of syringomatous adenomas is based on the histologic appearance of the lesion and its restriction to the dermis of the nipple [12]. Wide local excision to ensure free margins is the optimum treatment; the rate of recurrence is high in cases with involved margins [80, 81]. Syringomatous tumors of the nipple do not metastasize [82].

### Epidermal inclusion cyst

Also called an epidermoid cyst, this lesion is most common in the fourth decade of life [83]. This benign cutaneous or subcutaneous lesion arises from the proliferation or implantation of elements from the dermis (keratinized squamous epithelium) in a circumscribed space in the dermis to form a keratin-filled cyst [11]. Epidermal inclusion cysts can occur in any part of the body with hair follicles, but are very uncommon in the areolar region [83]. The clinical presentation consists of a superficial mobile nodule below the skin; on rare occasions, they can present with nipple discharge [84].

**Fig. 28:**
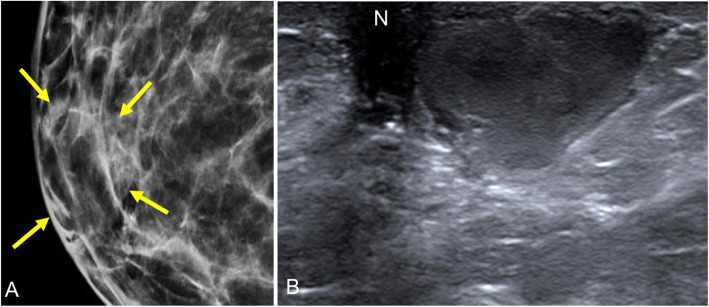
Epidermal inclusion cyst. A 37-year-old woman with a palpable retroareolar nodule in her right breast, without inflammation. **a** Synthesized 2D mammogram (craniocaudal view) shows a well-defined isodense nodule measuring 25 mm (arrows) adjacent to the nipple. **b** Ultrasound shows a well-defined hypoechogenic nodule below the skin of the right areola (*N* nipple)

On mammography, epidermal inclusion cysts appear as a well-defined superficial mass or a focal asymmetry in the retroareolar region. It is important for the mammographer to mark the palpable lesion on the skin before acquiring images. Ultrasound is the technique of choice for the diagnosis, showing an ovoid nodule with well-defined margins that can have a solid or complex cystic appearance with echogenic foci inside (related to keratin); the lesion has no internal Doppler signal and variable posterior acoustic enhancement (Fig. 28). Layered keratin deposition is seen as the “onion-ring sign,” alternating hyper- and hypo-echogenic concentric rings, and the “tram-track sign,” multiple alternating hyper- and hypo-echogenic lines [85, 86]. MRI shows a well-defined nodule with variable low-signal intensity on T2-weighted images, sometimes with an enhancing ring in postcontrast images [83]. If the cyst ruptures, its appearance can be indistinguishable from that of a malignant lesion, requiring biopsy specimens for histologic study [84, 87].

Complications of epidermal inclusion cysts include rupture, inflammation, infection (abscess), and even malignant transformation into squamous-cell carcinoma (0.045–12%, depending on the series) [83, 84]. Malignant transformation is more common in lesions greater than 5 cm [83, 84].

Cases with typical clinical and ultrasound findings require no treatment [83]. In these cases, biopsy is not recommended because the contents of the cyst can irritate the surrounding tissue and increase the risk of infection [57]. Some authors recommend surgical excision of lesions greater than 2 cm to avoid the risk of malignant transformation or of lesions that cause discomfort [83, 84, 87].

### Retroareolar cysts in adolescents

Retroareolar cysts in adolescents are caused by the obstruction and dilation of the terminal ducts that drain the Montgomery glands [88]. They can occur unilaterally or bilaterally. There are two main clinical types: inflammatory (more common) and asymptomatic [88, 89]. The inflammatory type presents as a palpable nodule associated with erythema and pain. The asymptomatic type presents as a palpable nodule without inflammatory signs or symptoms [88]. Occasionally, these cysts are associated with serous nipple discharge.

**Fig. 29:**
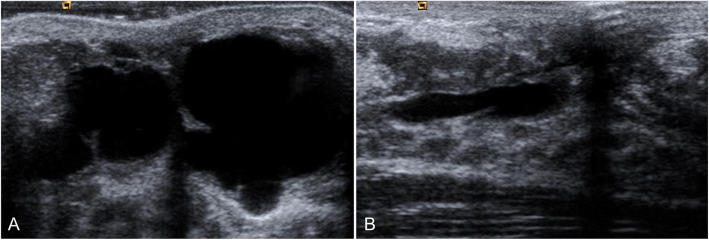
Retroareolar cyst in an adolescent. A 15-year-old girl presented with a palpable retroareolar mass in her left breast. Ultrasound shows a large thin-walled cystic lesion (**a**). Follow-up ultrasound 6 months later shows a marked decrease in the size of the lesion, which is oval and elongated (**b**)

Ultrasound is the technique of choice for both the initial assessment and follow-up. It shows one or more fine-walled oval cystic lesions measuring less than 20 mm (Fig. 29) [88]. Inflammatory cysts can contain debris, levels or septa, and increased peripheral Doppler signal. These cysts can be difficult to distinguish from a retroareolar abscess, but time helps; whereas retroareolar cysts have a benign clinical course and respond rapidly to oral antibiotics and nonsteroidal anti-inflammatory drugs, abscesses require drainage [89]. Symptomatic retroareolar cysts should be followed up for at least 7 days after initiating treatment to confirm the resolution of the lesion or a decrease in size; asymptomatic cysts resolve spontaneously, although ultrasound follow-up is recommended [88, 89].

### Gynecomastia

Gynecomastia is the most common breast disease in men [90]. It is caused by the non-neoplastic proliferation of the mammary ducts and stroma [91]. Multiple factors can favor gynecomastia, including physiological factors (high estrogen concentrations in newborns, adolescents, and the elderly), pharmacological factors, chronic disease (e.g., cirrhosis of the liver, chronic renal failure), genetic abnormalities (e.g., Klinefelter syndrome), hormonal disturbances (e.g., hyperthyroidism), tumors (e.g., Leydig cell tumors, Sertoli cell tumors, pituitary tumor), and obesity [90, 92].

The clinical history, physical examination, and imaging are important in the diagnostic workup of gynecomastia. The condition can be unilateral or bilateral; the most common clinical manifestation is the appearance of a painful (or burning) palpable retroareolar mass with increased breast volume [90]. Palpation reveals a soft, mobile, easily depressible, concentric retroareolar mass [92].

**Fig. 30:**
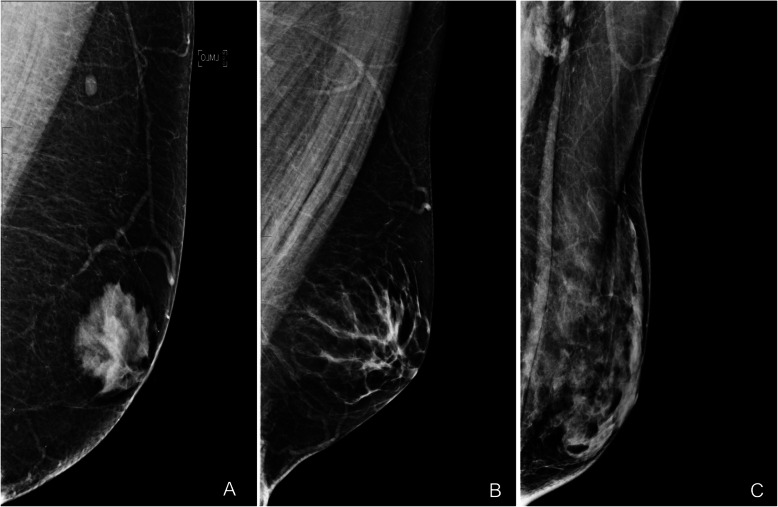
Radiologic patterns in gynecomastia. **a** Nodular. **b** Dendritic. **c** Diffuse

Three mammographic patterns have been described: nodular, dendritic, and diffuse (Fig. 30) [93, 94]. These patterns are related to histologic changes at different stages [90]. The nodular pattern is seen in the first year of the development of gynecomastia, and it is related to ductal and stromal proliferation. It is seen as a fan-shaped retroareolar density that radiates from the nipple that correlates with a hypoechogenic nodule with circumscribed margins on ultrasound. If the stimulus provoking gynecomastia is removed, the condition is reversible [92]. The dendritic pattern is seen more than 1 year after the onset, and it is related to fibrotic changes in the stroma and dilation of the ducts. Mammography and ultrasound both show finger-shaped prolongations radiating from the nipple to the retroareolar region. The diffuse pattern appears like a combination of the nodular and dendritic patterns or similar to a heterogeneous dense pattern in the female breast [94]. The diffuse pattern is associated with exposure to external estrogens [90]. On rare occasions, the imaging findings cannot differentiate gynecomastia from malignancy and biopsy is required for histologic study.

## Malignant disease

### Paget’s disease of the nipple

**Fig. 31:**
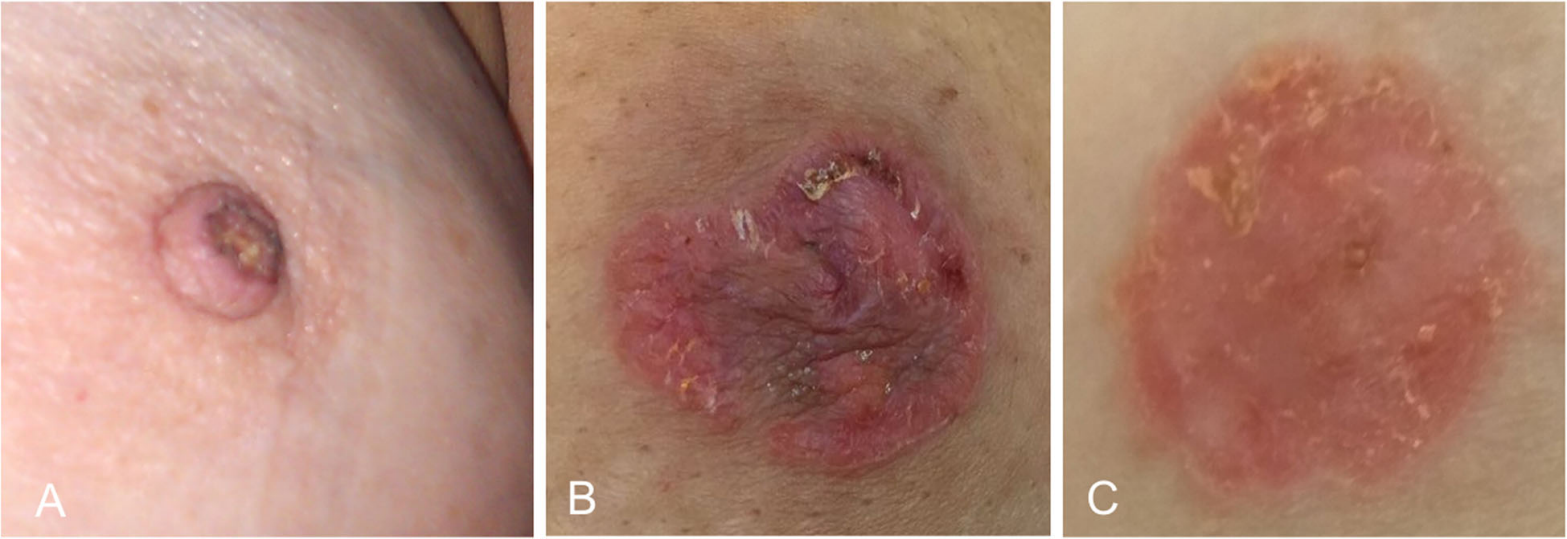
Cutaneous manifestations of Paget’s disease of the nipple. **a** Crusted ulcerated papule in the center of the nipple. **b** Scaly erythematous plaque with erosions that destroys the nipple. **c** Scaly erythematous plaque that covers the entire nipple

Defined by the presence of neoplastic cells (Paget cells) in the epidermis of the nipple [4], Paget’s disease of the nipple comprises 1% to 3% of all breast carcinomas, being most common in the fifth and sixth decades of life [29]. This entity is suspected clinically. It manifests with erythema, erosion, and ulceration of the nipple, which is sometimes associated with a palpable retroareolar mass and/or nipple retraction or discharge (Fig. 31) [33]. The definitive diagnosis requires skin biopsy and histologic study.

**Fig. 32:**
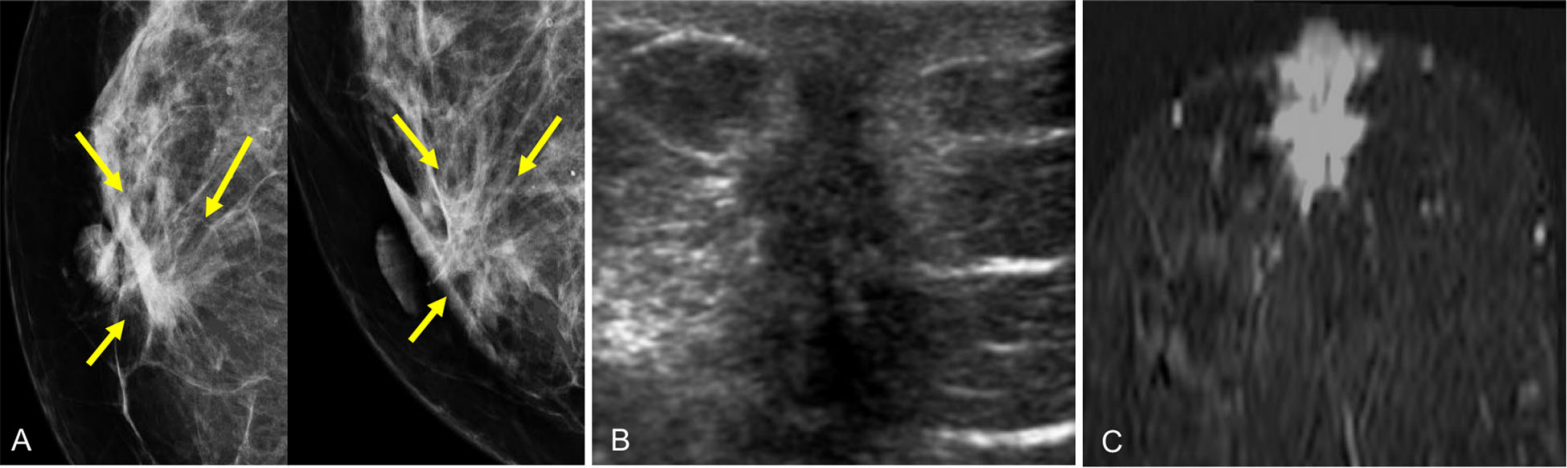
Paget’s disease (I). A 56-year-old woman with right nipple retraction. **a** 2D mammogram shows a spiculated retroareolar mass in the right breast with nipple retraction and skin thickening; **b** on ultrasound, it is seen as a solid lesion with ill-defined borders. **c** MRI shows the retroareolar lesion extending to the nipple-areolar complex. Histologic study revealed infiltrating ductal carcinoma extending to the epidermis

**Fig. 33:**
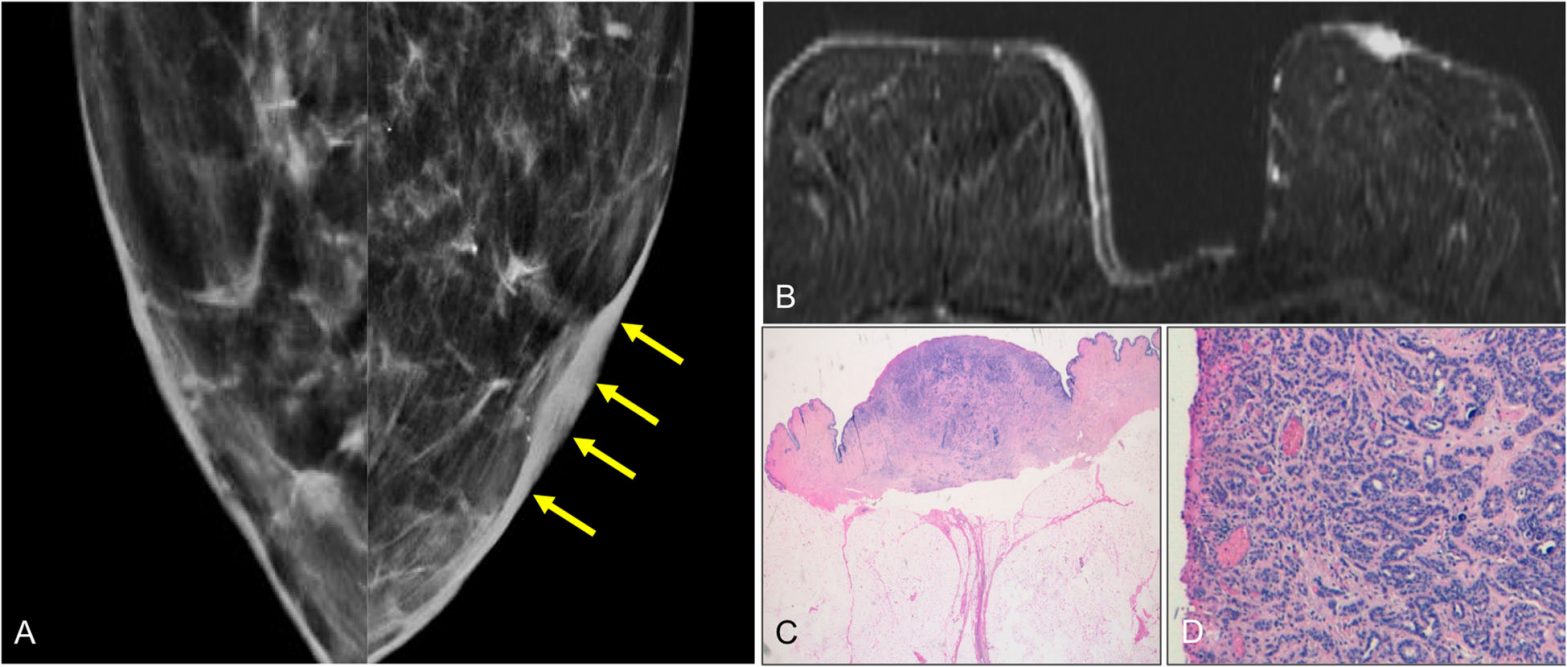
Paget’s disease (II). An 81-year-old woman with an ulcerated lesion in her left nipple. **a** Synthesized 2D mammogram shows slight skin thickening in the left nipple-areolar complex (arrows). **b** MRI shows differential pathologic enhancement of the left nipple-areolar complex. **c**, **d** Hematoxylin-eosin-stained punch-biopsy specimen (× 0.3 and × 4, respectively) shows ductal carcinoma with extensive infiltration of the nipple and ulceration of the epidermis (Paget’s disease)

Imaging plays a fundamental role in the study of the extent of disease and in the therapeutic management, because 90% of cases also have an underlying ductal carcinoma in situ or infiltrating ductal carcinoma [29, 33]. Mammography is the initial imaging test. The findings include skin thickening, a retroareolar mass, or pleomorphic calcifications. The mass can be seen in the subareolar region or more removed from the nipple-areolar complex. Nevertheless, the mammogram is normal in up to 50% of cases [28]. Ultrasound is usually done after mammography to confirm the findings (Fig. 32). MRI is very important for determining the extent of disease in patients with negative mammography and ultrasound findings who are candidates for conservative surgery (Fig. 33) [28, 29]. MRI shows abnormal uptake of contrast material, which can be non-masslike or masslike in relation to associated ductal carcinoma in situ or invasive ductal carcinoma [33]. Conservative surgery has proven a viable alternative to mastectomy in selected patients with Paget’s disease [29, 95, 96].

### Ductal carcinoma in situ

Ductal carcinoma in situ is considered a precursor that might develop into invasive cancer. By definition, it is a noninvasive malignant cellular proliferation contained within a duct by the basement membrane [11]. Generally, ductal carcinoma in situ is detected on screening mammograms in asymptomatic patients; these lesions can involve the nipple-areolar complex, in most cases by intraductal extension.

**Fig. 34:**
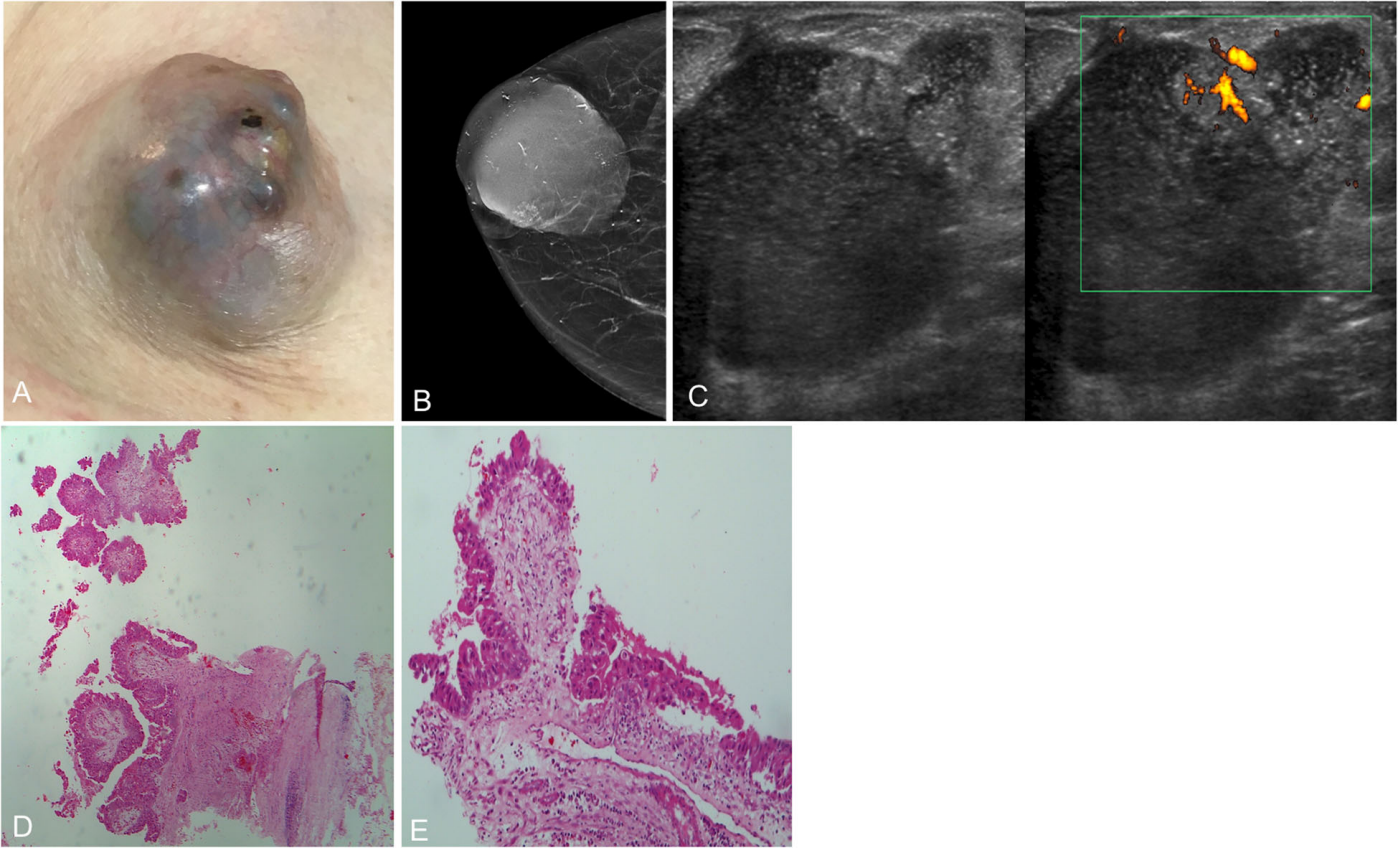
Ductal carcinoma in situ. **a** Photograph of an 85-year-old woman with a large palpable retroareolar mass with marked involvement of the right nipple-areolar complex. **b** Synthesized 2D mammogram shows a circumscribed retroareolar mass with associated pleomorphic calcifications. **c** Ultrasound shows a cystic mass with debris inside and a solid mural nodule with a penetrating vessel in the power-Doppler study. **d** Hematoxylin-eosin stain (× 10). Fragment of the cyst wall with slight chronic inflammatory involvement and vascular congestion and epithelial lining made up of few layers of markedly pleomorphic elements and dense eosinophilic cytoplasm. Note the proliferation of the papillary pattern, with fronds with wide fibroconnective stems lined with similar pleomorphic elements (intraductal carcinoma within the cyst). **e** Close-up (Hematoxylin-eosin stain × 20)

On mammography, the most characteristic findings are microcalcifications of variable morphology (present in 75–90%), although it can also present as a solid mass or even as architectural distortion [97, 98]. On ultrasound, ductal carcinoma in situ is usually not seen, though it can manifest as a slightly hypoechogenic solid mass within a duct or within the parenchyma, extending to and dilating an adjacent duct in the retroareolar region (Fig. 34) [99]. MRI is more sensitive in the detection of ductal carcinoma in situ (especially intermediate- and high-grade lesions) and is more accurate in evaluating its extent and in planning treatment [100–102]. The most common manifestation is non-masslike enhancement distributed segmentally or linearly with an internal pattern of clumped enhancement [99].

### Invasive ductal carcinoma

**Fig. 35:**
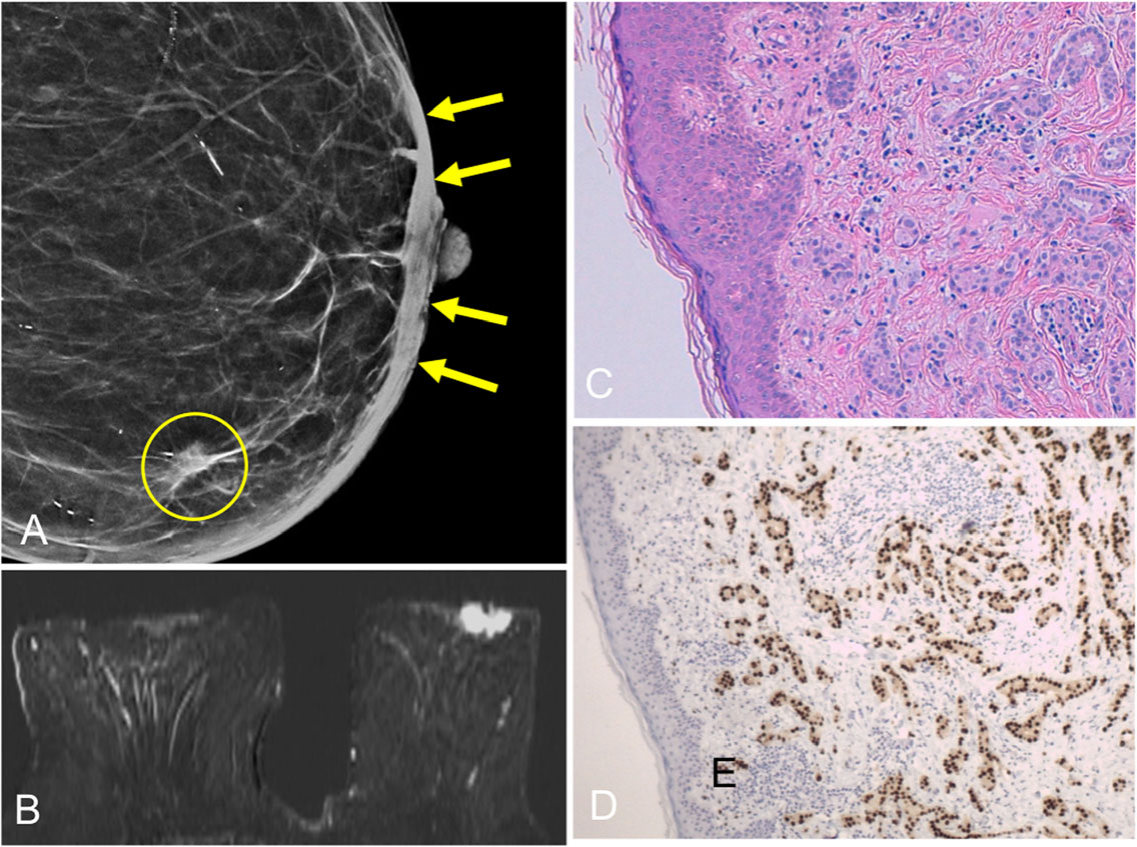
Invasive ductal carcinoma (I). An 88-year-old woman with a palpable nodule in the lower inner quadrant of her left breast. **a** Synthesized 2D mammogram shows a spiculated nodule (circle), classified as infiltrating lobular carcinoma at histology. Note the thickening of the skin and of the nipple-areolar complex (arrows). **b** MRI shows pathologic asymmetric enhancement of the left nipple-areolar complex. **c** Hematoxylin-eosin stain of punch-biopsy specimen (× 10) shows extensive dermal infiltration by infiltrating ductal carcinoma. **d** Immunohistochemistry (× 4) shows diffuse nuclear expression of estrogen receptors. These are two synchronous tumors

Invasive ductal carcinoma is the most common malignant tumor of the breast. Occasionally, it can be located immediately behind the nipple or it can originate in another location and extend to the nipple (Fig. 35). Nearly 10% arise in the central ducts less than 2 cm from the nipple [32].

**Fig. 36:**
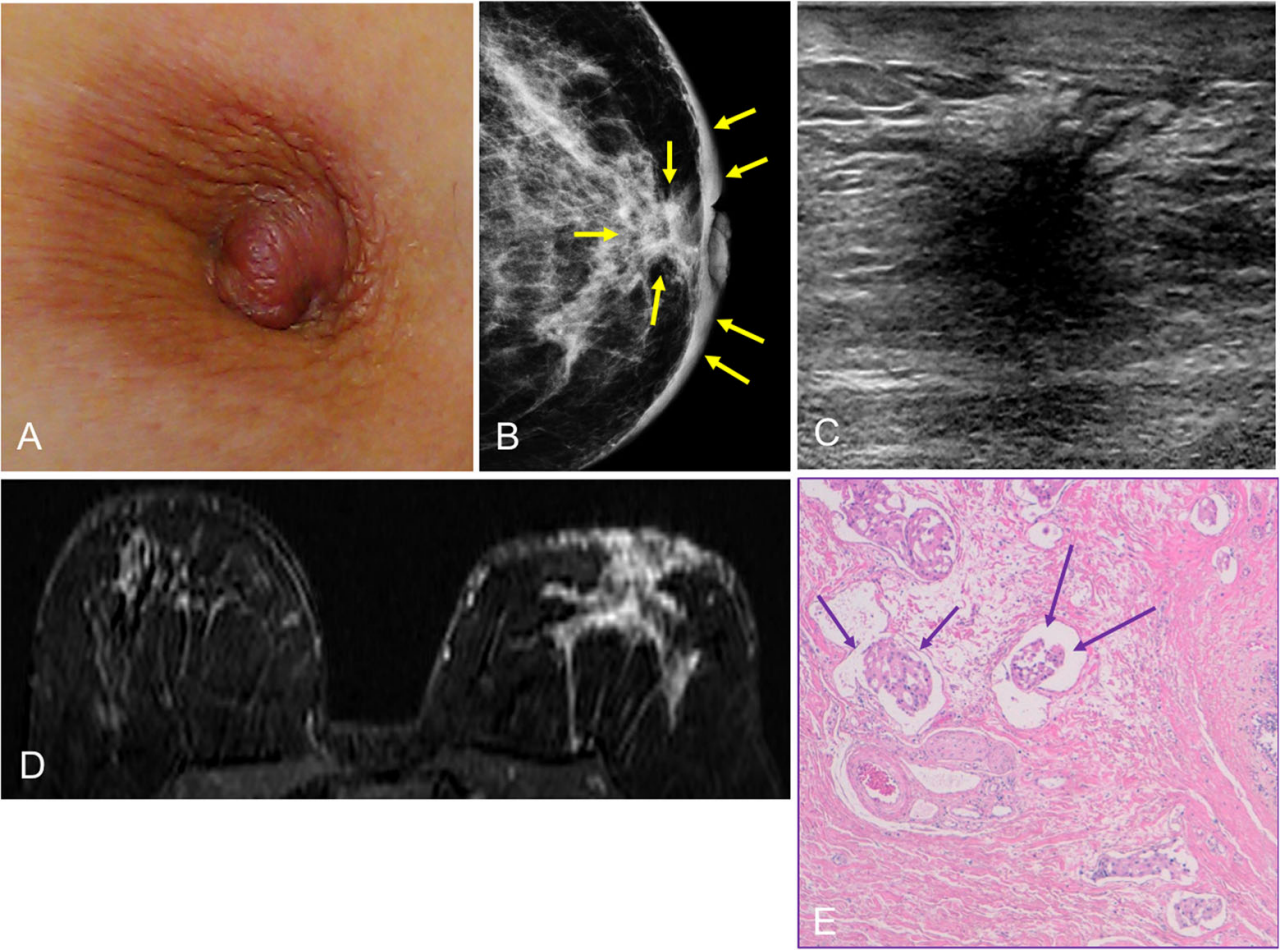
Invasive ductal carcinoma (II). **a** Photograph of a 54-year-old woman with left nipple retraction. **b** Mammogram shows skin thickening and increased retroareolar density; **c** on ultrasound, the lesion is hypoechogenic and ill-defined. **d** MRI shows a large lesion in the left breast involving the nipple-areolar complex. **e** Hematoxylin-eosin stain (× 4) shows lymphatic invasion of the dermis of the nipple by infiltrating ductal carcinoma (arrows)

In cases involving the nipple-areolar complex, the most common clinical manifestation is unilateral nipple retraction and distortion of the areola (Fig. 36). It is important to differentiate between inversion and retraction of the nipple. Inversion refers to the complete invagination of the nipple, which is mostly symmetrical and physiological. Retraction refers to focal inversion of the nipple-areolar complex and is asymmetrical and acquired. Both inversion and retraction can have benign or malignant causes; the time course and the presence of underlying disease are important [5].

Invasive ductal carcinoma generally presents as an ill-defined retroareolar mass [4]. In cases of clinically suspected invasive ductal carcinoma with negative findings at mammography and ultrasound, MRI should be done. Sometimes, the differential diagnosis must be done with papillary lesions or retroareolar abscesses. In case of doubt, the lesion should always be biopsied for histologic study.

### Radiation-associated angiosarcoma of the breast

**Fig. 37:**
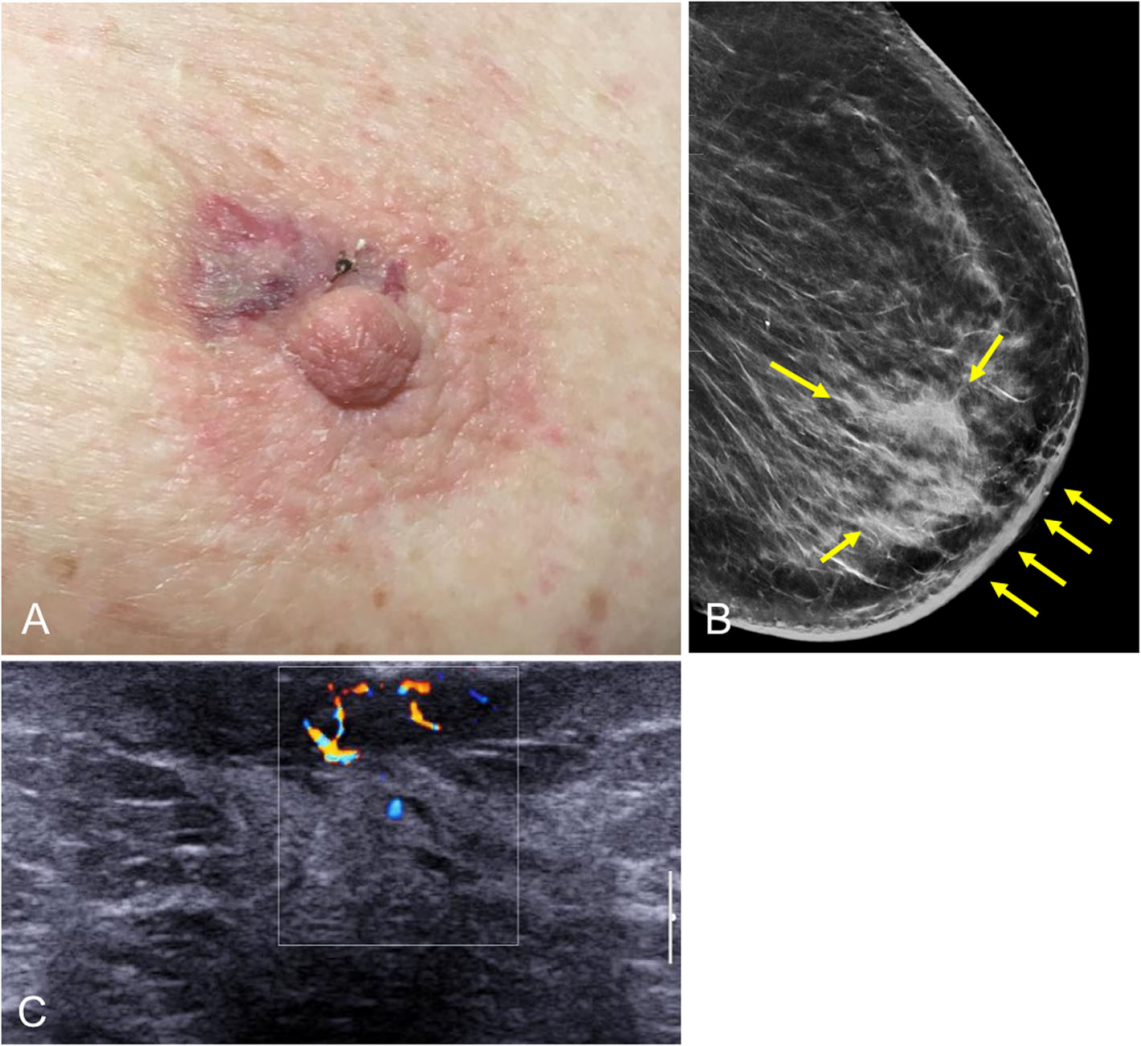
Radiation-associated angiosarcoma. A 76-year-old woman with a history of breast cancer treated with conservative surgery and whole-breast radiation therapy 6 years prior. **a** Photograph shows an ill-defined erythematous-violaceous infiltrated plaque with an eroded area occupying part of the areola. **b** Synthesized 2D mammogram shows skin thickening and interstitial edema in the retroareolar region of the left breast. **c** Ultrasound shows an ill-defined hypoechoic skin lesion with an internal Doppler signal. Punch biopsy diagnosed radiation-associated angiosarcoma, and the patient underwent mastectomy

Radiation-associated angiosarcoma of the breast is an uncommon aggressive malignant process that develops in women who have received radiation therapy for previous breast cancer [103]. This condition is becoming more common with increased use of conservative surgery, which always involves adjuvant whole-breast radiotherapy [104]. Radiation-associated angiosarcoma develops 5 to 7 years after radiation therapy and mainly affects older women. It can affect any part of the breast, including the nipple-areolar complex (Fig. 37) [105]. Patients present with areas of ecchymosis or skin thickening that mimic bruises or hematomas, sometimes delaying diagnosis [105, 106].

The findings on mammography and ultrasound are nonspecific (skin thickening, skin retraction, parenchymal distortion) and can even remain occult due to changes occurring after conservative treatment. Normally, skin thickening and breast density tend to decrease markedly 2 years after radiation therapy. It is very important to suspect this entity and obtain skin biopsies in patients with skin changes and a history of radiation therapy in the same breast [106]. MRI shows skin thickening (seen better in T2-weighted images) with rapid uptake in contrast-enhanced T1-weighted imaging and pathological nipple enhancement; up to 25% of cases have an associated intraparenchymal mass [106, 107]. The main role of MRI is to determine the extent of the process. Treatment consists of wide local resection or mastectomy. The prognosis is poor; metastases normally develop, especially in the lungs [106, 108].

### Diagnostic imaging algorithm

**Fig. 38:**
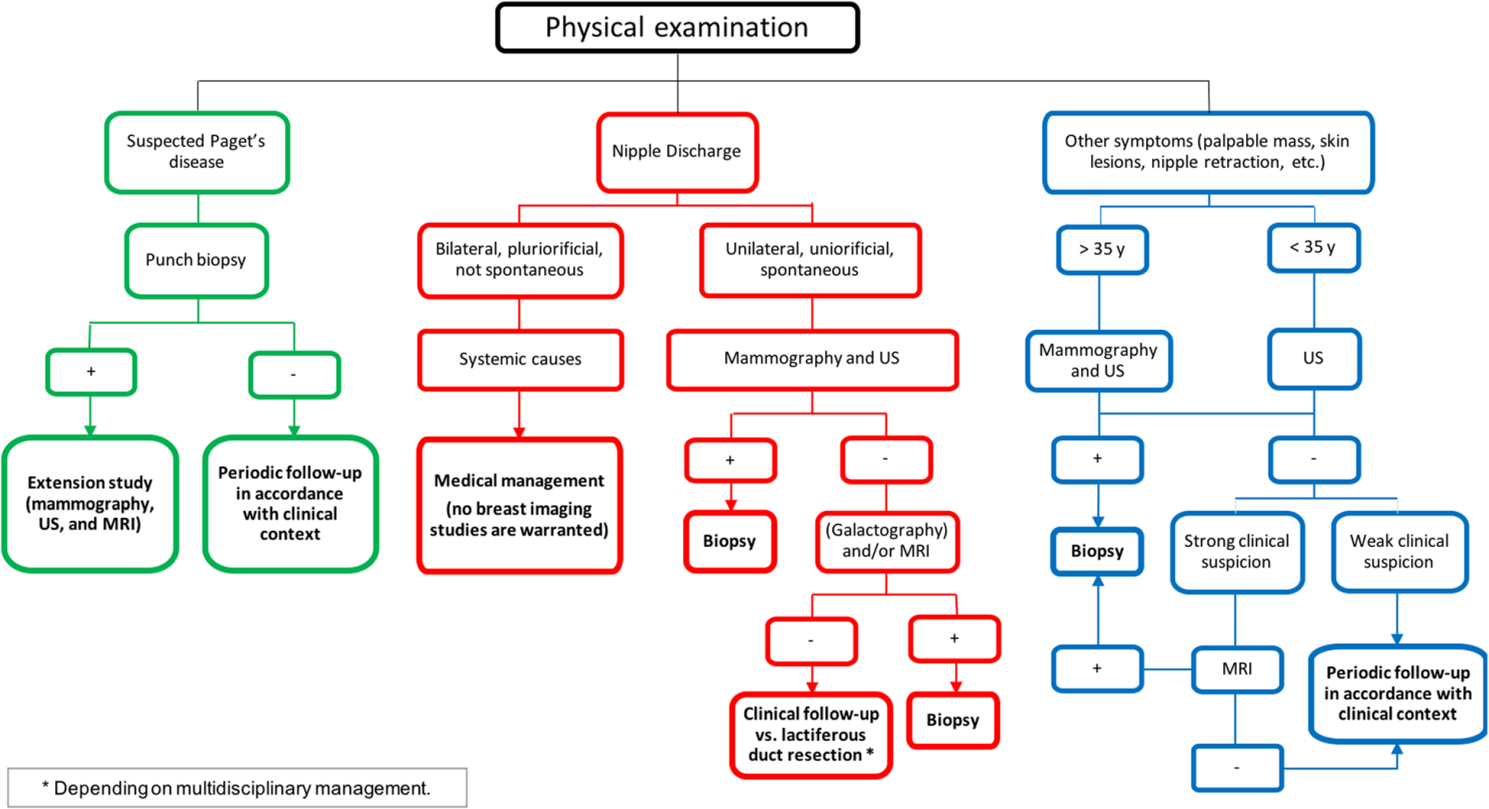
Diagnostic imaging algorithm

The management of nipple disease needs to begin with a thorough physical examination to direct the imaging workup based on the patient’s signs and symptoms. Figure 38 shows the diagnostic imaging algorithm used at our center.

## Conclusion

Imaging studies play an essential role in the diagnostic workup of conditions involving the nipple-areolar complex. The anatomic complexity of the nipple-areolar complex requires a specific, multimodal approach to imaging evaluation. Radiologists must be accustomed to meticulous management of the different imaging modalities. Many conditions involving the nipple-areolar complex have nonspecific clinical and radiological presentations that can delay diagnosis. It is essential to evaluate the clinical, radiological, and histological findings together to establish an accurate diagnosis.

## Data Availability

Data sharing is not applicable to this article as no datasets were generated or analyzed during the current study.

## Abbreviations

MRI: Magnetic resonance imaging
TDLU: Terminal ductal lobular unit
